# Ref-1 drives ulcerative colitis induced systemic defects in hematopoietic cells

**DOI:** 10.1038/s42003-026-09860-z

**Published:** 2026-03-19

**Authors:** Ramesh Kumar, Rahul Kanumuri, Sarah S. Burns, Baskar Ramdas, Lakshmi Reddy Palam, Santhosh Kumar Pasupuleti, Xuepeng Wang, Rajaraman Eri, Kulmira Nurgali, Mark R. Kelley, Reuben Kapur

**Affiliations:** 1 Department of Pediatrics, Herman B Wells Center for Pediatric Research, Indiana University School of Medicine, Indianapolis, IN USA; 2 School of Science, STEM College, RMIT University, Melbourne, VIC Australia; 3 Institute for Health and Sport, Victoria University, Melbourne, VIC Australia

**Keywords:** Stem cells, Gastroenterology

## Abstract

Ulcerative colitis (UC) is a debilitating, immune-mediated inflammatory disorder of the gastrointestinal (GI) tract with far-reaching consequences on distal organs, including the bone marrow. Here, we describe the molecular mechanisms that contribute to UC-induced abnormal hematopoiesis. We show that chronic UC drives HSPC differentiation toward myelopoiesis in an APE1/Ref-1/HIF-1α/IL-1r1-dependent manner. Blockade of the redox-activity of APE1/Ref-1 with APX3330 inhibits the elevated expression of HIF-1α in HSPCs and reverses the aberrant HSPC dynamics under the inflammatory *milieu* of UC, including suppression of pro-inflammatory Ly6C^hi^ monocytes. Using echinomycin, we pharmacologically blocked HIF-1α activity and found that HIF-1α mediates inflammatory responses via downstream IL-1r1 signaling. Blockade of the redox activity of ref-1 rescues the abnormal HSPC function. Our data highlight the significance of the APE1/Ref-1/HIF-1α/IL-1r1 signaling cascade in aberrant hematopoiesis that contributes to the pathophysiology of chronic UC through a feed-forward loop.

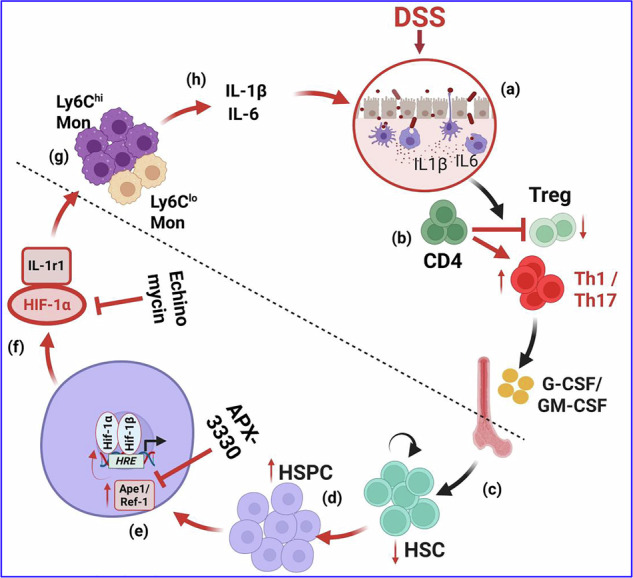

## Abstract (web summary)

Chronic ulcerative cholitis contributes to the differentiation of HSPCs toward myelopoiesis in an APE1/Ref-1/HIF-1α/IL-1r1 dependent manner. Blockade of the redox activity of Ref-1 with APX3330 rescues the abnormal HSC function.

## Introduction

Ulcerative colitis (UC) is a chronic, recurring inflammatory disease of the gastrointestinal (GI) tract. While UC can occur across all ages, studies suggest that approximately 20% of patients with UC present before the age of 20 years, and the incidence rate continues to rise in the United States of America^1,2^. The etiopathogenesis of UC development is unclear; however, accumulating data suggest a role for the pathological immune response against microbial and environmental antigens in genetically predisposed individuals^3,4^. It appears that both the innate and adaptive immune systems, in response to microbiota, are involved in maintaining intestinal homeostasis. Studies have advocated that aberrant immune cells and their cytokines are thought to increase intestinal permeability by disrupting intestinal epithelial integrity^5,6^.

All the blood cells and immune cells are consistently replenished lifelong by a rare population of hematopoietic stem cells (HSCs) in a hematopoietic hierarchical manner in the bone marrow (BM)^7^. HSCs must maintain self-renewal and differentiation potential over time to sustain hematopoiesis. In the steady state, HSCs are relatively quiescent; however, inflammatory conditions induced by infection or stress can activate HSCs to meet the demand of the total blood cell pool, a process termed “emergency hematopoiesis^8^.” The fate of HSCs’ renewal and differentiation is tightly controlled by both cell-intrinsic and cell-extrinsic factors^9^. Under stress-induced tissue homeostasis, infection, and inflammatory conditions, HSCs can give rise to various lineage-restricted hematopoietic stem and progenitors (HSPCs) through the pool of intermediate hematopoietic cells called multipotent progenitors (MPPs). HSPCs are further stepwise differentiated into blood cell subtypes, including granulocytes, monocytes, and lymphocytes, which participate in the host immune response^10–12^. The interaction between evolutionarily conserved pathogen-associated molecular patterns (PAMPs) of bacteria and viruses and pathogen recognition receptors (PRRs), e.g., Toll-like receptors (TLRs) and the nucleotide-binding oligomerization domain-like receptors (NLRs) expressed by HSPCs may lead to “emergency myelopoiesis^13–16^.” Infection-induced chronic inflammation with a variety of pathogenic organisms, such as bacteria, viruses, and parasites, can result in profound alterations in hematopoiesis in the BM, including expansion of the early progenitor compartment (Lineage^−^c-Kit^+^ Sca1^+^; also called LSK cells), changes in HSC differentiation and long-term reconstitution properties, and migration patterns ^17–20^.

Recently, gut microbiota have emerged to play a critical role in the manifestation and maintenance of many diseases, including UC, rheumatic arthritis, metabolic syndrome, neurodegeneration, and malignancy^21^. However, several studies suggest that intestinal microbiota serve as a key regulator of hematopoiesis, as germ-free and antibiotic-treated mice presented increased pathogen burden and a decrease in the HSPC pool^22–24^, suggesting some of the responses associated with diverse infections are context dependent, which likely reflects different host–pathogen interactions.

Microbial infections drive pro-inflammatory cytokines, interferons (IFN-α/γ), tumor necrosis factor (TNF)-α, interleukin (IL)-1, IL-6, growth factors (G-CSF/M-CSF)^25–27^. These pro-inflammatory cytokines and bacterial endotoxins (e.g., LPS) released by leaky and inflamed gut enter into systemic circulation, causing inflammatory stress conditions in the BM^28^. However, it remains largely unknown how pathophysiologic conditions of chronic ulcerative colitis contribute to hematopoiesis, what underlying molecular mechanism (s) are involved in its regulation, and if the hematopoietic defects can be corrected. Thus, at a more systems level, how signals originating in the gut are translated into activation of cells of the innate and adaptive immune systems, as well as those in the BM that give rise to these cells at a very primitive level, remains unknown. Importantly, whether these systemic, tissue-based global changes can be reversed is unknown.

To address these questions, we used a chronic colitis mouse model that recapitulates several key features of UC in humans. Our focus was to delineate the mechanisms underlying chronic ulcerative colitis (cUC)-driven aberrant hematopoiesis and to emphasize strategies to mitigate it. Our data demonstrates that the APE1/Ref-1/HIF-1α/IL-1r1 signaling pathway plays an important role in aberrant hematopoiesis.

## Results

### APE1/Ref-1 inhibitor, APX3330, rescues cUC-mediated hematopoietic defects

**Fig. 1:**
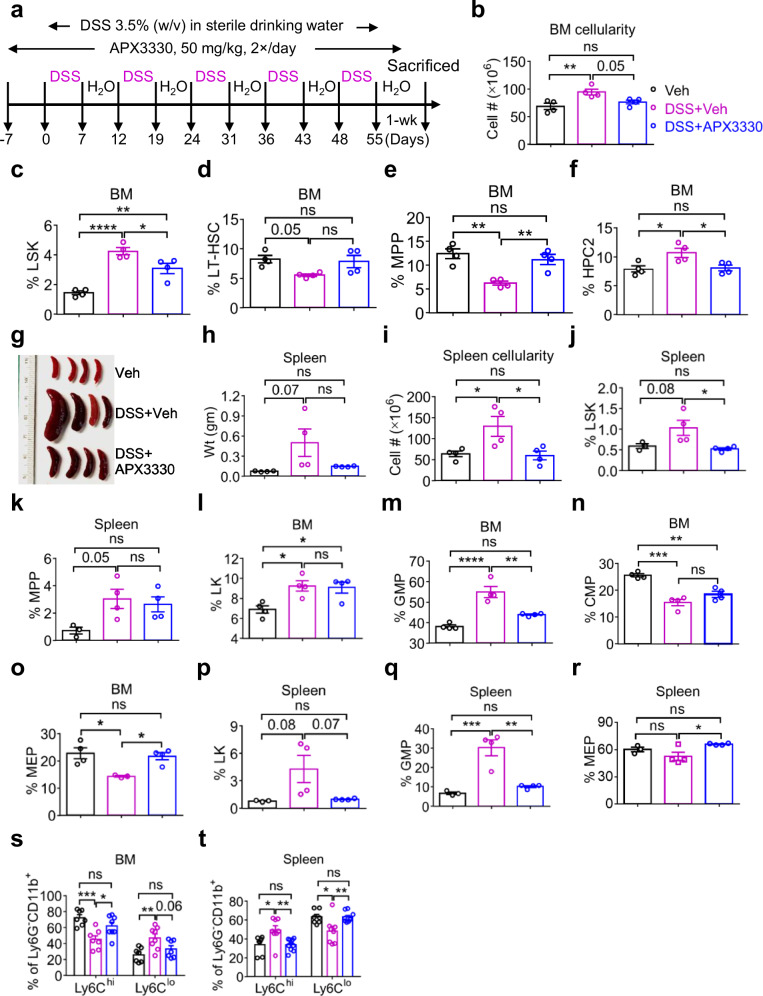
APE1/Ref-1 inhibitor, APX3330, rescues cUC-mediated hematopoietic defects. **a** Experimental scheme illustrating the treatment protocol for the APX3330 drug and DSS (3.5%, w/v). Mice were gavaged with APX3330 at 50 mg/kg body weight twice daily. The treatment regimen was initiated 7 days before DSS and continued throughout the DSS cycles. **b** Shows the total number of bone marrow (BM) cells, **c** shows the frequency of LSK cells (Lin^−^Sca-1^+^c-Kit^+^), **d** shows the frequency of LT-HSCs (Lin^−^Sca-1^+^c-Kit^+^CD150^+^CD48^−^), **e** shows the frequency of MPPs or ST-HSCs (Lin^−^Sca-1^+^c-Kit^+^CD150^−^CD48^−^), and (**f)** shows the frequency of myeloid-biased hematopoietic progenitor (HPC2) cells (Lin^−^Sca-1^+^c-Kit^+^CD150^+^CD48^+^). **g** Shows representative images of spleen from Veh, DSS + Veh, and APX3330 + DSS treated mice, **h** spleen weight, (**i**) the total number of splenocytes, **j** the frequency of LSK cells, **k** the frequency of MPP cells, **l** the frequency of LKs (Lin^−^Sca-1^−^c-Kit^+^), **m** the frequency of granulocyte-macrophage progenitors, GMPs (Lin^−^Sca-1^−^c-Kit^+^CD16/32^+^CD34^+^), **n** the frequency of common-myeloid progenitors, CMPs (Lin^−^Sca-1^−^c-Kit^+^CD16/32^lo^CD34^+^), **o** the frequency of megakaryocyte-erythrocyte progenitors, MEPs (Lin^−^Sca-1^−^c-Kit^+^CD16/32^−^CD34^−^) in the BM of Veh, DSS+Veh, and APX3330 + DSS treated mice. **p** the frequency of LKs, **q** the frequency of GMPs, and (**r**) the frequency of MEPs in the spleen of Veh, DSS + Veh, and APX3330 + DSS treated mice. Frequencies of Ly6C^hi^ and Ly6C^lo^ monocytes characterized by Ly6G; CD11b+; and Ly6C hi and lo expression in the BM (**s**) and spleen (**t**) of Veh, DSS + Veh, and APX3330 + DSS treated mice. Results are either representative data of two independent experiments (**b–r;** *n* = 3–4 mice per group) or aggregate data of two independent experiments (**s**, **t;** *n* = 7–8 mice per group), and each dot represents an individual mouse. Data are shown as mean ± SEM. Statistical significance was determined using one-way ANOVA (**b–r**) or two-way ANOVA (**s**, **t**) with Tukey’s multiple comparison test. *P < 0.05, **P < 0.01, ***P < 0.001, and ****P < 0.0001; n.s., not significant.

Inflammatory signaling arises from functional interactions between the gut microbiota and the host immune system and contributes to disease pathophysiology not only in the intestine but also in distant organs, including the lungs, liver, and BM^29–31^. Oxidative stress is a key factor closely linked to the development and progression of inflammation in UC^32^. In this context, apurinic/apyrimidinic endonuclease 1/reduction oxidation factor-1 (APE1/Ref-1; also called ref-1) protein regulates oxidative stress by modulating reduction-oxidation (redox) activities of various transcription factors, which are known to be involved in cell survival and inflammation^33^. We hypothesized that chronic inflammatory conditions in UC contribute to abnormal hematopoiesis and that blockade of APE1/Ref-1 redox activity by its specific inhibitor, APX3330, would reverse cUC-induced hematopoietic defects. APX3330 is an orally administered small-molecule inhibitor that has completed Phase I clinical trials for safety and toxicity in adult cancer patients who have failed all other standard treatments (NCT03375086)^34–36^. APX3330 demonstrated excellent safety and pharmacokinetic/pharmacodynamic (PK/PD) profile, and tissue biopsy evaluations indicated reduced transcriptional activity of key regulators of cancer survival pathways, including HIF1, NF-κB, and STAT3, indicating that APX3330 mediates the redox activity of the ref-1 protein target as expected. Subsequent to this trial, a phase IIb trial in diabetic retinopathy using APX3330 through Ocuphire Pharma was successfully completed (NCT04692688). APX3330 is unique in that it provides anti-inflammatory and neuroprotective effects, as evidenced by previous studies^37^. Therefore, we hypothesized that oral administration of APX3330 could reverse abnormal hematopoiesis and confer protection against UC with minimal side effects. To this end, we established a DSS-induced cUC mouse model, as shown in Fig. S1a and described in the “Materials and Methods”. Among the DSS regimens tested, administration of 3.5% DSS over 5 cycles of consecutive 7 days in each cycle most effectively induced UC-associated hematopoietic defects (Fig. S1b–l). In contrast, lower concentrations (2–3%) of DSS did not reproducibly induce hematopoietic defects. To evaluate the long-term impact of cUC on hematopoiesis and the role of oxidative stress, we treated mice with the ref-1 inhibitor APX3330. APX3330 was administered by oral gavage at 50 mg/kg body weight twice daily, beginning one week prior to DSS treatment and continuing through the final DSS cycle, while control mice received vehicle (veh) alone (Fig. 1a and “Materials and Methods”). One week after completion of the final DSS cycle, mice were sacrificed for analysis. cUC mice exhibited significantly increased BM cellularity (Figs. 1b and S1b) and elevated frequency of LSK cells (Lin^−^Sca-1^+^c-Kit^+^) (Figs.1c and S1d). Analysis of HSC subsets, as shown in Fig. S1e, showed reduced frequencies of long-term hematopoietic stem cells (LT-HSCs; LSK^gated^CD48^−^CD150^+^) (Figs. 1d and S1f) and multipotent progenitors (MPPs; LSK^gated^CD48^−^CD150^−^); also referred to as short-term hematopoietic stem cells (ST-HSCs) (Figs. 1e and S1g), and increased frequency of myeloid-biased hematopoietic progenitor cell-2 (HPC-2; LSK^gated^CD48^+^CD150^+^) (Figs. 1f and  S1h) in the BM of cUC mice compared to control mice. Absolute numbers of LSKs, LT-HSCs, and HPC-2 (Fig. S1i, j, l–o) were increased in the BM of cUC mice compared to control mice. In contrast, APX3330-treated cUC mice showed correction in BM cellularity (Fig. 1b), frequencies of LSKs, LT-HSCs, MPPs and HPC-2 cells in the BM compared to veh-treated control mice (Fig. 1c–f). The APX3330 treatment also restored absolute numbers of LSKs, LT-HSCs, and HPC-2 cells in the BM of cUC mice (Fig. S1m–o). APX3330 treatment did not affect steady-state hematopoiesis in veh-treated control mice (Fig. S2a–i). Given the pronounced splenomegaly observed in cUC mice (Figs. 1g–i and S3a–c), we next assessed whether this phenotype reflected extra-medullary hematopoiesis (EMH). Indeed, spleens from cUC mice showed an enhanced expansion in the frequencies of LSKs (Figs. 1j and S3d), LT-HSCs (Fig. S3e), MPPs (Fig. 1k), and HPC-2 cells (Fig. S3g) and in the absolute numbers of LSKs, LT-HSCs, MPPs, and HPC-2 cells (Fig. S3h–o) compared to spleens from control mice, indicating cUC can lead to EMH in the spleen^13^. Most importantly, the cUC-induced spleen abnormalities, such as spleen weight and cellularity, frequency of LSKs (Fig. 1g–j), and altered absolute numbers in HSC subsets (Fig. S3l–o) were corrected by treatment of these mice with APX3330.

Under steady-state conditions, inflammatory signaling is required to maintain HSPC pool size and support myelopoiesis; however, sustained inflammation is detrimental in diseased settings. Accordingly, we analyzed c-Kit^+^ progenitor populations and observed increased frequencies of LKs (Lin^−^c-Kit^+^) (Figs. 1l and S4b) and granulocyte-monocyte progenitors (GMPs; LK^gated^CD16/32^hi^CD34^+^) (Figs. 1m and S4c), along with reduced frequencies of common-myeloid progenitors (CMPs; LK^gated^CD16/32^low^CD34^+^) (Figs. 1n and S4d), megakaryocyte-erythroid progenitors (MEPs; LK^gated^CD16/32^−^CD34^−^) (Fig. 1o and S4e) and common-lymphoid progenitors (CLPs; Lin^−^CD127^+^c-Kit^lo^Sca-1^lo^) (Fig. S4f) in the BM of cUC mice. Consistently, absolute numbers of LKs and GMPs (Figs. S4g, h and S5a, b) were increased, whereas CMPs and CLPs (Figs. S4i, j and S5c) were decreased in the BM of cUC mice compared to control mice. In the spleen, cUC mice exhibited increased frequencies of LKs (Figs. 1p and S4k) and GMPs (Figs. 1q and S4l), as well as elevated absolute numbers of LKs, GMPs, and CMPs (Fig. S4m–o), consistent with enhanced extramedullary myelopoiesis. Similar to the corrections observed in the HSC compartment, APX3330 treatment normalized the frequencies of GMPs and MEPs (Fig. 1m–o) and restored absolute numbers of LKs, GMPs, and CLPs (Fig. S5a–c) in the BM. In the spleen, APX3330 treatment also corrected the frequencies of LKs, GMPs, and MEPs (Fig. 1p–r) and reduced the absolute numbers of LKs and GMPs (Fig. S5d, e) in cUC mice compared to veh-treated control mice.

To assess whether cUC contributes to HSC/HSPC survival, differentiation, or apoptosis, we assessed apoptosis and cell-cycle status within the LSK compartment. Flow cytometric analysis using Annexin V and 7-AAD staining revealed a greater than 2-fold reduction in apoptosis among LSK cells from cUC mice compared to controls (Fig. S6a, b). Subsequent cell-cycle analysis by DAPI (4’,6-diamidino-2-phenylindole) staining demonstrated increased proportions of LSK cells in the G0/G1 and S-phase in cUC mice (Fig. S6c, d), indicating that cUC contributes to both enhanced survival and increased cell-cycle progression of LSK cells.

**Fig. 2:**
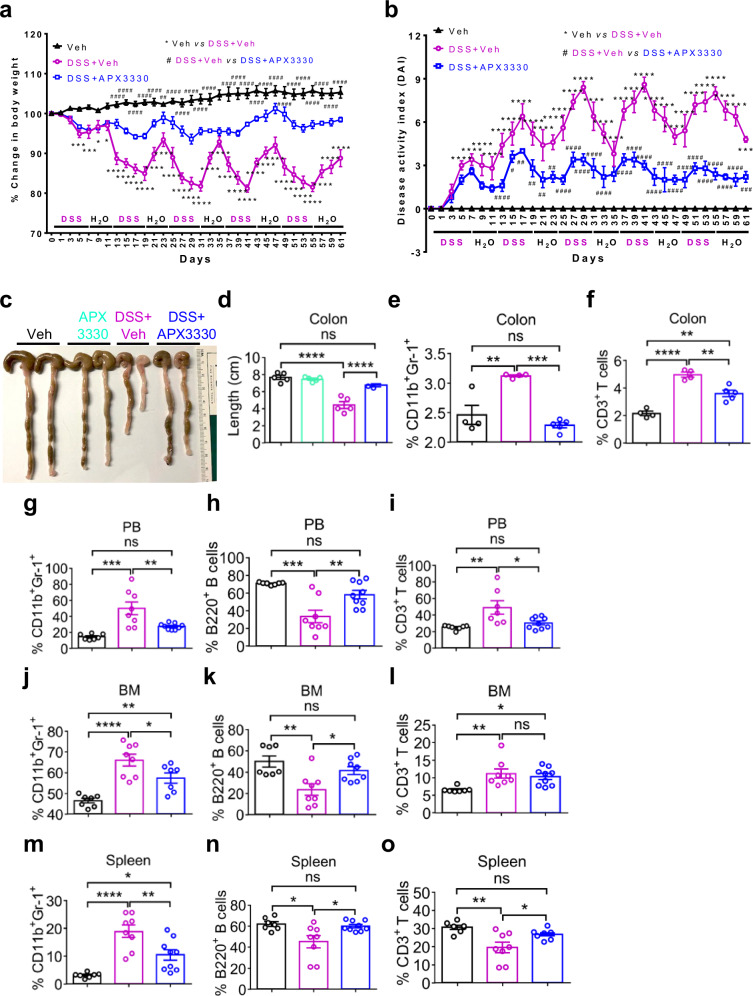
APE1/Ref-1 inhibitor, APX3330, attenuates cUC by normalizing immune cell populations. **a** Shows the changes in body weight, **b** changes in disease activity index (DAI) over five cycles of DSS treatment, **c** representative images of colon tissue, and (**d)** the quantification of colon length (in cm) in Veh, DSS + Veh, and APX3330 + DSS treated mice. **e** Shows the frequency of CD11b^+^Gr1^+^ neutrophils, and (**f)** the frequency of CD3^+^ T cells in the colon of Veh, DSS + Veh, and APX3330 + DSS treated mice. Frequencies of neutrophils (CD11b^+^Gr-1^+^) (**g**), B220^+^ B cells (**h**), and CD3^+^ T cells (**i**) in the peripheral blood (PB) of Veh, DSS+Veh, and APX3330 + DSS treated mice. Frequencies of neutrophils (CD11b^+^Gr-1^+^) (**j**), B220^+^ B cells (**k**), and CD3^+^ T cells (**l**) in the BM of Veh, DSS+Veh, and APX3330 + DSS treated mice. Frequencies of neutrophils (CD11b^+^Gr-1^+^) (**m**), B220^+^ B cells (**n**), and CD3^+^ T cells (**o**) in the spleen of Veh, DSS+Veh, and APX3330 + DSS treated mice. Results are either representative data of two independent experiments (**a–f**; *n* = 4 mice per group) or are the cumulative data of two independent experiments (**g–o**; *n* = 7–8 mice per group), and each dot represents an individual mouse. Data are shown as mean ± SEM. Statistical significance was determined using two-way ANOVA **(a, b, d**) or one-way ANOVA (**g–o**) with Tukey’s multiple comparison test. ^*^P < 0.05, ^**^P < 0.01, ^***^P < 0.001, and ^****^P < 0.0001; n.s., not significant.

To understand whether the hematopoietic defects observed in the BM and spleen were driven by cUC and whether APX3330 treatment confers protection against intestinal damage, we determined circulating pro-inflammatory Ly6C^hi^ monocytes, which play a critical role in colonic tissue homeostasis in both human UC and murine models^38–40^. We observed a reduced frequency of Ly6C^hi^ monocytes in the BM and a concomitant increase in the spleen of cUC mice, whereas APX3330 treatment restored the balance between Ly6C^hi^ and Ly6C^lo^ monocytes in both the BM and spleen of cUC mice (Fig. 1s, t). These findings indicate that APX3330 treatment suppresses the emigration of Ly6C^hi^ monocytes and protects mice from colonic tissue damage^41^. Consistent with these hematopoietic effects, DSS-challenged mice displayed features of cUC, including body weight loss (Figs. 2a and S7a), increased disease activity index (DAI; Figs. 2b and S7b), and shortened colon length (Figs. 2c, d and S7c–e). APX3330 treatment significantly ameliorated all of these disease parameters. Moreover, DSS treatment induced a pronounced inflammatory immune response in the colon (Fig. S7f–m), which was markedly attenuated by APX3330 treatment. Specifically, APX3330 reduced the accumulation of pro-inflammatory CD11b^+^Gr-1^+^ myeloid and CD3^+^ T cells in the colon (Fig. 2e, f), as well as CD11b^+^Gr-1^+^ neutrophils, B220^+^ B cells, and CD3^+^ T cells in the PB (Fig. 2g–i), BM (Fig. 2j–l) and spleen (Fig. 2m–o) of cUC mice. Collectively, these data demonstrate that chronic inflammatory conditions in cUC drive expansion of the HSPC pool size and bias differentiation toward myelopoiesis through a feed-forward inflammatory loop. Pharmacological inhibition of ref-1 redox activity by APX3330 restores cUC-induced hematopoietic defects and mitigates intestinal pathology, suggesting that correction of inflammation-induced hematopoietic defects may protect against the development of UC.

### APX3330 rescues cUC-induced defects via inhibiting G-CSF and hypoxia-inducible transcription factor-1α (HIF-1α)

**Fig. 3:**
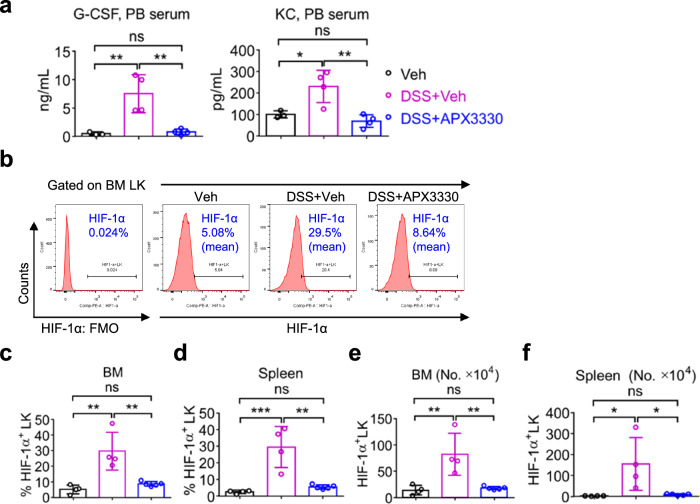
APX3330 rescues cUC-induced defects via inhibiting G-CSF and hypoxia-inducible transcription factor-1α (HIF-1α). **a** Quantification of cytokine G-CSF (ng/mL) and chemokine KC (pg/mL) in the peripheral blood serum from Veh, DSS+Veh, and APX3330 + DSS treated mice. **b** Flow-cytometric profile showing gating strategy for HIF-1α^+^ LK cells using intracellular staining. Frequencies of HIF-1α^+^ LK (Lin^−^Sca-1^−^c-Kit^+^) cells from the BM (**c**), and spleen (**d**); the absolute numbers of HIF-1α^+^ LK (Lin^−^Sca-1^−^c-Kit^+^) cells from the BM (**e**), and spleen (**f**) of Veh, DSS + Veh, and APX3330 + DSS treated mice. For fluorescent minus one (FMO) staining, an equal concentration of fluorophore-matched isotype control antibody was used. Results are from 4 mice in each experimental group. Data are shown as mean ± SEM. Statistical analysis was performed using a one-way ANOVA with Tukey’s multiple comparison test. ^*^P < 0.05, and ^**^P < 0.01; n.s., not significant.

In subsequent analyses, we performed multiplex serum cytokine profiling of peripheral blood from mice treated with veh or APX3330, with or without DSS. Expression levels of 31 cytokines and chemokines covering a broad spectrum of immune and inflammatory mechanisms were quantified. Of these, 11 cytokines/chemokines (G-CSF, IL-1β, IL-6, IL-10, IP-10, IL-17, MCP-1, MIG, MIP-1α, MIP-1β, and TNF-α) were significantly elevated in cUC mice compared to controls (Fig. S8a–k). Notably, granulocyte-colony stimulating factor (G-CSF) was the most abundantly increased cytokine in cUC mice (Fig. 3a, left panel). Elevated systemic G-CSF is known to promote HSPC mobilization from the BM by suppressing CXCL12/CXCR4 signaling in osteoblasts^42,43^. In addition, several chemokines involved in neutrophils and monocytes/macrophages recruitment, including KC (CXCL1) (Fig. 3a, right panel), IP-10 (CXCL10), MCP-1 (CCL2), MIG (CXCL9), MIP-1α (CCL3), MIP-1β (CCL4), and TNF-α (Fig. S8e–k) were among the most highly expressed factors in the serum of cUC mice^44^. Importantly, APX3330 treatment significantly reduced serum concentrations of G-CSF and KC in cUC mice relative to veh-treated controls (Fig. 3a), indicating that inhibition of ref-1 redox activity dampens systemic inflammatory signaling associated with cUC.

To identify molecular mechanisms driving accelerated myelopoiesis in cUC, we interrogated the expression of NF-ĸB in HSPCs, as APE1/Ref-1 regulates the redox-sensitive DNA-binding activity of NF-ĸB^33^. However, intracellular flow cytometric analysis did not detect phospho-NF-ĸBp65^+^ LKs in either the BM or spleen of cUC mice (data not shown). Given that G-CSF stabilizes and activates hypoxia-inducible factor-1 α (HIF-1α) even under normoxic conditions^45^, we next hypothesized that elevated G-CSF in cUC mice promotes HIF-1α activation in HSPC cells and that APX3330 administration suppresses this response. Consistent with this model, cUC mice exhibited a marked increase in both the frequency and absolute number of HIF-1α-expressing LK cells in the BM and spleen, which was significantly reduced by APX3330 treatment compared to veh-treated control mice (Fig. 3b–f). Altogether, these data indicate that cUC induces HIF-1α expression in HSPCs through an APE1/Ref-1/G-CSF-dependent mechanism, driving HSPCs differentiation toward granulopoiesis. In contrast, pharmacologic inhibition of ref-1 redox activity attenuates G-CSF-mediated HIF-1α activation in HSPCs and mitigates cUC-associated hematopoietic dysregulation.

### The blockade of redox activity of APE1/Ref-1 by APX3330 improves the repopulating capacity of cUC-triggered defective HSCs

**Fig. 4:**
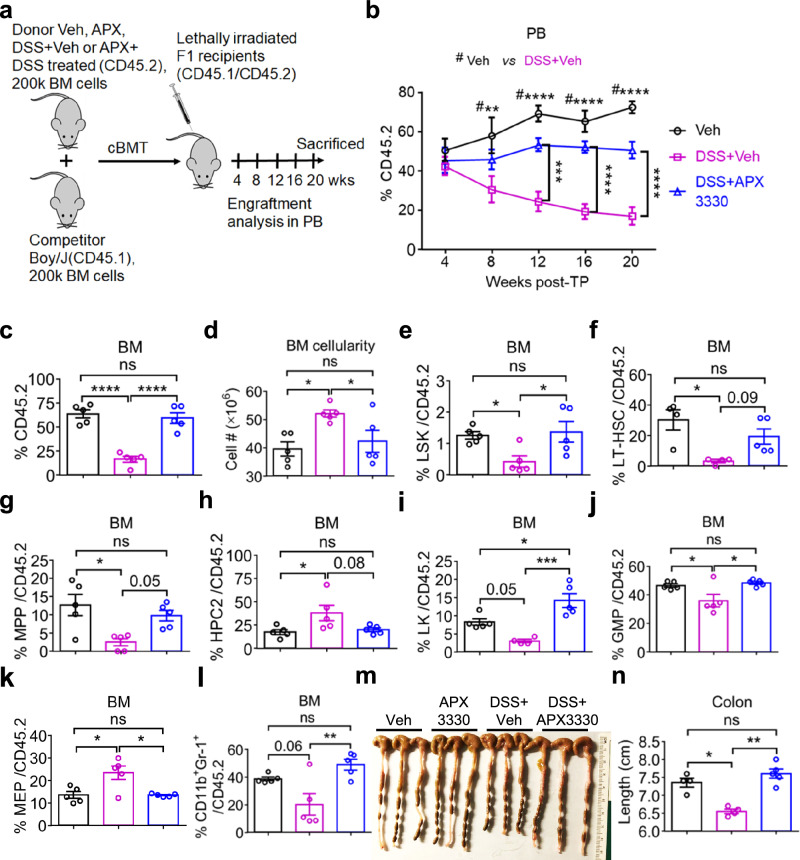
The blockade of redox activity of APE1/Ref-1 by APX3330 improves the repopulating capacity of cUC-triggered defective HSC under transplant settings. **a** A schematic presentation of primary competitive bone-marrow transplantation (cBMT) assay. **b** Analysis of PB at 4, 8, 12,16, and 20 weeks post-cBMT, and BM (**c**) at 20-week post-cBMT showing engraftment of donor-derived CD45.2 cells. **d** Shows the total BM cells in Veh, DSS+Veh, and APX3330 + DSS mice. The frequencies of donor-derived LSK cells (**e**), LT-HSCs (**f**), MPPs (**g**), and HPC2 (**h**) in the BM of F1 recipient mice of the indicated genotypes following treatment with Veh, DSS or APX3330. Frequencies of donor-derived LKs (**i**), GMPs (**j**), and MEP progenitors (**k**) in the BM of F1 recipient mice of the indicated genotypes following treatment with Veh, DSS or APX3330. **l** Shows the frequency of donor-derived neutrophils (CD11b^+^Gr1^+^) cells in the BM of F1 recipient mice of the indicated genotypes following treatment with Veh, DSS or APX3330. **m** Shows images of colon tissues, and (**n)** quantification of colon length (cm) in F1 recipient mice of the indicated genotypes following treatment with Veh, DSS or APX3330. The results are from 5 mice in each experimental group. Data are presented as mean ± SEM. Statistical significance was determined using either two-way ANOVA (**b)** or one-way ANOVA (**c–l**, **n)** with Tukey’s multiple comparison test for the analysis of differences between the experimental groups. ^*^P < 0.05, ^**^P < 0.01, ^***^P < 0.001, and ^****^P < 0.0001; n.s., not significant.

Given that cUC impairs the repopulating capacity of HSCs (Fig. S9b–d), we postulated that treatment with APX3330 could rescue the repopulating capacity of HSCs. To test this, we performed a competitive BMTP assay using the whole BM cells of veh control, cUC (DSS + veh), and cUC mice treated with APX3330 (APX3330 + DSS), as outlined in Fig. 4a. Surprisingly, PB chimerism of CD45.2^+^ donor cells derived from APX3330-treated cUC mice showed sustained improvement in repopulating capacity from week 4 through week 20 post-transplantation; whereas, CD45.2^+^ donor cells from veh-treated cUC mice exhibited a marked reduction in repopulating capacity starting at week 4 post-transplantation, which continued to decline throughout the engraftment period (Fig. 4b). At 20 weeks post-transplantation, recipient mice were sacrificed to assess HSC chimerism and their competence to differentiate into the various cell lineages. Consistent with the restoration of PB chimerism, the APX3330 treatment rescued the BM chimerism of HSCs derived from cUC mice to levels comparable to those of veh-treated control mice (Fig. 4c). Notably, CD45.2^+^ donor cells derived from APX3330-treated cUC mice also rescued BM cellularity (Fig. 4d) and frequencies of LSKs (Fig. 4e), LT-HSCs (Fig. 4f), MPPs (Fig. 4g), and HPC-2 cells (Fig. 4h) in the BM. Likewise, the frequencies of LKs (Fig. 4i), GMPs (Fig. 4j), MEPs (Fig. 4k), and CD11b^+^Gr-1^+^ neutrophil cells (Fig. 4l) were also rescued. To interrogate whether CD45.2^+^ donor cells from cUC had any effects on the colon restoration, we quantified the respective colons in Fig. 4m and found that recipient mice transplanted with CD45.2^+^ donor cells of cUC displayed significantly reduced colon length (Fig. 4n). Surprisingly, recipients of CD45.2^+^ donor cells from APX3330-treated cUC mice exhibited restored colon length comparable to that observed in recipients of veh-treated control donor cells (Fig. 4m, n). These findings indicate that APX3330 treatment rescued the gut in an HSC-autonomous manner. Collectively, under transplantation conditions, our data demonstrate that ref-1 mediates its effects through the regulation of G-CSF secretion and HIF-1α activity. Pharmacological inhibition of ref-1 redox activity by APX3330 improves HSC function and corrects the pathophysiological conditions of chronic UC.

### HIF-1α-specific inhibitor, echinomycin, reduces the accumulation of proinflammatory CD4^+^ T helper 1 (Th1) and Th17 cells in the colon by reducing IL-1β and IL-6 production

Next, we aimed to delineate the role of HIF-1α signaling downstream of APE1/Ref-1 using the HIF-1α-specific inhibitor, echinomycin^46–48^. To this end, we divided B6 mice into 3 groups: veh, DSS+veh, and DSS + Echin. DSS and echinomycin (10 µg/kg body weight, administered by intraperitoneal (i.p.) injection on alternate days) were delivered according to the experimental scheme as shown in Fig. 1a. Consistent with the effects observed following APX3330 treatment, echinomycin administration significantly restored body weight, DAI score, colon length, splenomegaly, and spleen cellularity in cUC mice to levels comparable to those of veh-treated controls (Fig. S10a–f).

Given that the recruitment of Ly6C^hi^ monocytes to the inflamed colon regulates T cell responses, including pathogenic Th1 and Th17 cells^49,50^, we next examined the effects of echinomycin on CD4^+^ T cell responses. We observed that echinomycin administration normalized the frequencies of CD4^+^ T cells, Treg cells, colitogenic Th1 and Th17 cells, as well as the Treg/CD4^+^ T cell ratio in the colons of cUC mice (Fig. S10g–k). In addition, echinomycin treatment significantly reduced the frequencies of CD11b^+^ myeloid cells and CD3^+^ T cells expressing the proinflammatory cytokines IL-1β and IL-6 (Fig. S10l–m). We also observed that, in the colons of cUC mice, Th1 cells produced markedly elevated levels of GM-CSF (3.25-fold), while Th17 cells produced increased levels of both G-CSF (1.92-fold) and GM-CSF (4.07-fold); these increases were significantly attenuated by echinomycin treatment (Fig. S10n–p). Collectively, these findings indicate that pharmacological blockade of HIF-1α by echinomycin attenuates chronic UC by suppressing IL-1β and IL-6 production in CD11b^+^ myeloid and CD3^+^ T cells, thereby limiting colitogenic Th1 and Th17 cell responses.

### HIF-1α-specific inhibitor, echinomycin, restores cUC-induced defective hematopoiesis

**Fig. 5:**
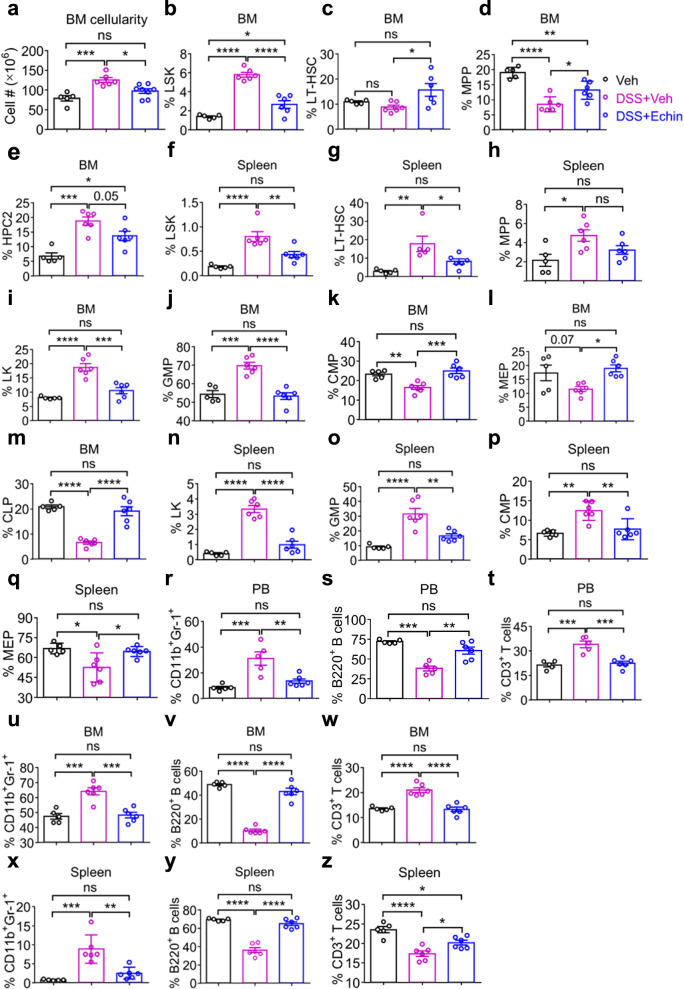
HIF-1α-specific inhibitor, echinomycin, restores cUC-induced defective hematopoiesis. **a** Shows the absolute BM cellularity in Veh, DSS + Veh, and DSS+Echin treated mice. **b** Shows the frequency of LSK cells in the BM of Veh, DSS+Veh, and DSS+Echin treated mice. Frequencies of (**c**) LT-HSCs, (**d**) MPPs, and (**e**) HPC2 within the LSK progenitors in the BM of Veh, DSS + Veh, and DSS + Echin treated mice. **f** Show the frequency of LSK cells in the spleen of Veh, DSS + Veh, and DSS + Echin treated mice. Frequencies of (**g)** LT-HSCs, and (**h)** MPPs within the LSK progenitors in the spleen of Veh, DSS +Veh, and DSS + Echin treated mice. **i** Shows the frequency of LK cells in the BM of Veh, DSS + Veh, and DSS + Echin treated mice. Frequencies of (**j)** GMPs, **k** CMPs, and (**l)** MEPs within the LK progenitors, and (**m)** the frequency of CLP (Lin^−^CD127^+^c-Kit^lo^Sca-1^lo^) in the BM of Veh, DSS + Veh, and DSS+Echin treated mice. **n** Shows the frequency of LK cells in the spleen of Veh, DSS + Veh, and DSS + Echin treated mice. Frequencies of (**o)** GMPs, **p** CMPs, and (**q)** MEPs within the LK progenitors in the spleen of Veh, DSS+Veh, and DSS+Echin treated mice. Frequencies of (**r)** CD11b^+^Gr1^+^ neutrophils, **s** B220^+^ B cells, and (**t)** CD3^+^ T cells in the PB of Veh, DSS + Veh, and DSS + Echin treated mice. Frequencies of (**u)** CD11b^+^Gr1^+^ neutrophils, (**v)** B220^+^ B cells, and (**w)** CD3^+^ T cells in the BM, and (**x)** CD11b^+^Gr1^+^ neutrophils, (**y)** B220^+^ B cells, and (**z)** CD3^+^ T cells in the spleen of Veh, DSS + Veh, and DSS + Echin treated mice. The results are from 5–6 mice in each experimental group. Data are shown as mean ± SEM. Statistical significance was determined using one-way ANOVA with Tukey’s multiple comparison test for the analysis of differences between the experimental groups. ^*^P < 0.05, ^**^P < 0.01, ^***^P < 0.001, and ^****^P < 0.0001; n.s., not significant.

Given that echinomycin treatment reduced G-CSF- and GM-CSF-producing Th1 and Th17 cells in the colons of cUC mice (Fig. S10n–p), we next assessed whether pharmacological blockade of HIF-1α by echinomycin would correct the defective hematopoiesis observed in cUC mice^51,52^. Consistent with the effects of APX3330, echinomycin treatment restored BM cellularity (Fig. 5a) and significantly corrected the frequencies and absolute numbers of LSKs, LT-HSCs, MPPs, and HPC-2 progenitors in both the BM (Figs. 5b–e and S11a–d) and spleen (Figs. 5f–h and S11e–h), compared with cUC mice.

Furthermore, echinomycin rescued the frequencies and absolute numbers of downstream hematopoietic progenitors, including LKs, GMPs, CMPs, MEPs, and CLPs in the BM (Figs. 5i–m and S11i–m) and spleen (Figs. 5n–q and S11n–q) of cUC mice. Similar to the corrections in the primitive hematopoietic compartments, the altered proportions of CD11b^+^Gr-1^+^ neutrophils, B220^+^ B cells, and CD3^+^ T cells in the PB (Fig. 5r–t), BM (Fig. 5u–w), and spleen (Fig. 5x, z) of cUC mice were also reversed following echinomycin treatment. Echinomycin did not inhibit steady-state hematopoiesis in control mice (Fig. S11r–x). Collectively, these data suggest that HIF-1α inhibition by echinomycin rescues cUC-induced aberrations in HSCs and HSPCs by re-establishing balanced myelopoiesis and lymphopoiesis.

### Echinomycin restores cUC-induced defective hematopoiesis by inhibiting Interleukin-1receptor1 (IL-1r1) signaling in HSPCs

**Fig. 6:**
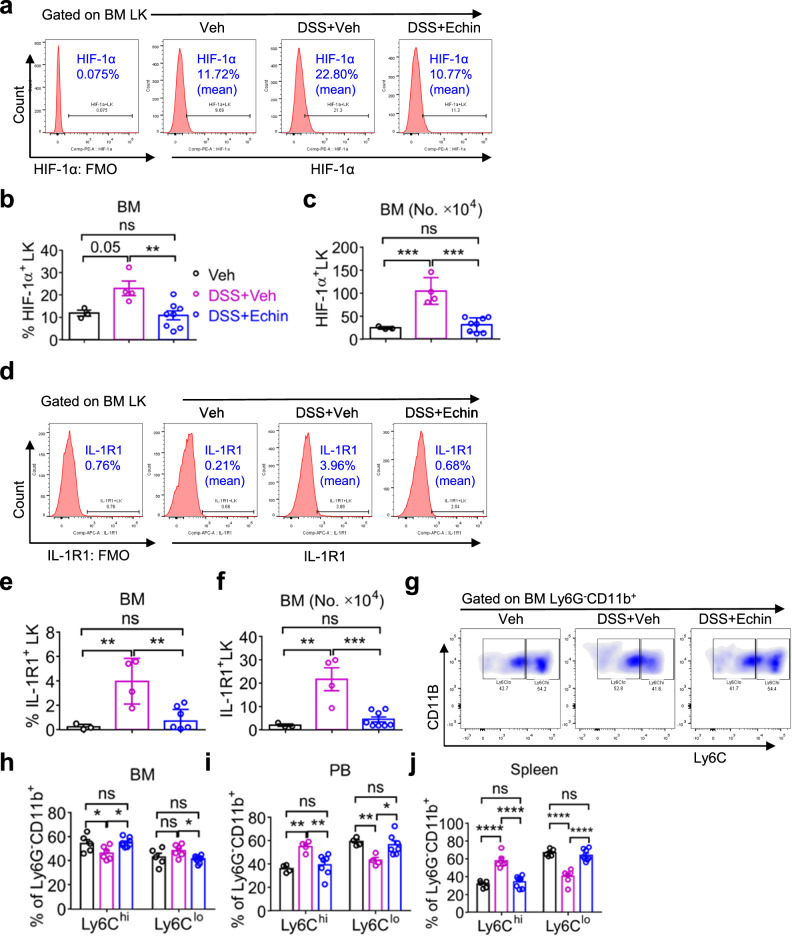
Echinomycin restores cUC-induced defective hematopoiesis by inhibiting Interleukin-1receptor1 (IL-1r1) signaling in HSPCs. **a** Flow-cytometric profile showing the gating strategy for HIF-1α^+^ LK cells in the BM of Veh, DSS + Veh, and DSS+Echin treated mice using intracellular staining. **b** The frequency of HIF-1α^+^ LK (Lin^−^Sca-1^−^c-Kit^+^) cells, and (**c**) the absolute number of HIF-1α^+^ LK (Lin^−^Sca-1^−^c-Kit^+^) cells in the BM of Veh, DSS+Veh, and DSS + Echin treated mice. **d** Flow-cytometric profile showing the gating strategy for IL-1r1^+^ LK cells in the BM of Veh, DSS + Veh, and DSS+Echin treated mice. **e** The frequency of IL-1r1^+^ LK (Lin^−^Sca-1^−^c-Kit^+^) cells, and (**f**) the absolute number of IL-1r1^+^ LK (Lin^−^Sca-1^−^c-Kit^+^) cells in the BM of Veh, DSS + Veh, and DSS + Echin treated mice. **g** Flow-cytometric profile showing the gating strategy for Ly6C^hi^ and Ly6C^lo^ cells in the BM of Veh, DSS + Veh, and DSS + Echin treated mice. The frequencies of Ly6C^hi^ and Ly6C^lo^ cells in the BM (**h**), PB (**i**), and spleen (**j**) of Veh, DSS + Veh, and DSS + Echin treated mice. The results are from 5 to 6 mice in each experimental group. Data are presented as mean ± SEM. Statistical significance was determined using either one-way ANOVA (**b**, **c**, **e**, **f**) or two-way ANOVA (**h–j**) with Tukey’s multiple comparison test for the analysis of differences between the experimental groups. ^*^P < 0.05, ^**^P < 0.01, ^***^P < 0.001 and ^****^P < 0.0001; n.s., not significant.

It is well established that IL-1r1 signaling plays a critical role in HSPC differentiation^53,54^. We therefore hypothesized that elevated HIF-1α expression in HSPCs regulates IL-1r1 signaling under the inflammatory conditions of cUC. To test this, cUC mice were treated with the HIF-1α inhibitor echinomycin, which significantly reduced HIF-1α expression in immature LK progenitors compared with veh-treated cUC mice (Fig. 6a–c). To determine whether echinomycin also modulated IL-1r1 expression, we performed IL-1r1 surface staining. IL-1r1 expression was markedly increased on LKs in the BM of cUC mice and was significantly reduced following echinomycin compared with veh-treated cUC mice (Fig. 6d–f).

IL-1r1 signaling is initiated upon binding of its ligands IL-1α, IL-1β, and IL-1 receptor antagonist (IL-1Ra)^55^. The IL-1β/IL-1R1 signaling axis is known to promote the recruitment of inflammatory monocytes to inflamed tissues, including the colon^56–58^, and global loss of IL-1 signaling impairs infiltration of both Ly6C^hi^ and Ly6C^lo^ monocytes in inflammatory settings^56^. In line with these observations, echinomycin administration, similar to APX3330 treatment, restored the balance between pro-inflammatory Ly6C^hi^ and anti-inflammatory Ly6C^lo^ monocytes in cUC mice (Fig. 6g–j). Based on these findings, we hypothesized that *IL-1r1* deficiency would confer protection against DSS-induced cUC. To test this, we performed competitive BM transplantation assays and observed that recipient mice transplanted with *IL-1r1* KO donor cells displayed normal engraftment of CD45.2^+^ cells in both the PB (Fig. S12a, b) and BM (Fig. S12c), compared to recipients transplanted with WT CD45.2^+^ cells and treated with DSS. Notably, the usual expansion of myeloid cells seen in mice transplanted with WT cells and treated with DSS was not seen in *IL-1r1* KO recipients treated with DSS (Fig. S12d, e). Moreover, the reduction in the colon length upon DSS treatment of *IL-1r1* KO bearing recipients was only modest compared to controls (Fig. S12f, g). Consistent with reduced intestinal inflammation, *IL-1r1* KO recipient mice exhibited a markedly lower frequency of pathogenic Th17 cells in the colon following DSS treatment compared with WT CD45.2^+^ cell-bearing recipients (Fig. S12h). Collectively, these data demonstrate that HIF-1α promotes inflammatory signaling through the IL-1r1 pathway, driving aberrant HSPC differentiation under cUC conditions. Pharmacological inhibition of HIF-1α by echinomycin attenuates IL-1r1-mediated inflammation and restores hematopoietic homeostasis under cUC conditions.

## Discussion

UC is a debilitating, immune-mediated chronic inflammatory disorder of the GI tract that arises from dysregulated innate and adaptive immune responses to the gut microbiota^3–5^. Here, we demonstrate that chronic UC (cUC) profoundly remodels the hematopoietic hierarchy, leading to aberrant progenitor expansion, stem cell dysfunction, and pathological myelopoiesis. Our findings identify that an APE1/Ref-1-depedent redox signaling axis involving HIF-1α and downstream IL-1r1 signaling is a central driver of inflammation-induced hematopoietic dysregulation and a potential therapeutic target (graphical abstract).

Although interactions among the gut microbiota, intestinal barrier function, and immunity have been extensively studied in health and disease^4,21,59^, the mechanisms by which cUC-associated inflammation contributes to hematopoiesis remain poorly understood. Given the central role of oxidative stress in UC pathogenesis, we hypothesized that ref-1-mediated redox signaling promotes inflammation-driven hematopoietic defects. Ref-1 mediates redox-dependent activation of transcription factors, including HIF1, NF-κB, and STAT3^32,33^. APX3330, a selective inhibitor of the redox activity of ref-1 protein, offers excellent anti-inflammatory properties^34–37^. We therefore further hypothesized that oral administration of APX3330 would reverse cUC-induced hematopoietic defects and protect mice from UC development. The current study examined the mechanisms driving aberrant hematopoiesis and its restoration to normal under cUC conditions using the DSS-induced colitis model and the ref-1 inhibitor, APX3330. We show that mice challenged with DSS recapitulate several key features of UC, including increased weight loss, diarrhea, rectal bleeding, and abnormal immune responses typically observed in humans with UC^59,60^. We show that cUC mice exhibit an increased frequency of LSK cells that contain HSPCs, driven by enhanced cell survival and cell-cycle progression. cUC resulted in a markedly reduced frequency of LT-HSCs and MPPs, accompanied by an increased frequency of myeloid-biased HPC-2 progenitors in the BM. Notably, although the frequency of LT-HSCs was reduced, their absolute numbers were increased, likely reflecting overall BM hypercellularity rather than preservation of stem cell integrity. We further show that oral administration of APX3330 (50 mg/kg, twice daily) effectively corrected these defects, restoring the balance of the HSC pool. This study provides evidence that pharmacological inhibition of ref-1 redox activity normalizes HSC dynamics under chronic inflammatory conditions, supporting a role for APE1/Ref-1-mediated redox signaling in cUC-associated hematopoietic dysfunctions. We further show that cUC leads to progressive splenomegaly, which is directly linked to the extra-medullary hematopoiesis, EMH^8,61,62^. APX3330 administration corrected splenomegaly, as evidenced by reduced spleen weight, decreased spleen cellularity, and restoration of primitive HSC pools. These findings indicate that ref-1 redox signaling contributes not only to BM dysfunction but also to pathological hematopoiesis in peripheral organs during chronic intestinal inflammation.

Previous studies have demonstrated that microbiota imbalance, antibiotic treatment, germ-free conditions, and microbial products (e.g., LPS) can directly regulate HSPC expansion, mobilization, and differentiation toward granulocytes in a TLR2- and/or TLR4/MyD88-dependent manner^22,63,64^. Consistent with these observations, we show that increased BM cellularity in cUC mice was driven by GMP expansion at the expense of CLPs and MEPs within the HSPC compartment, which correlated with increased serum G-CSF levels. Systemic G-CSF release promotes HSPC mobilization to the periphery by suppressing CXCR4/CXCL12 signaling in osteoblasts^65^. By inhibiting ref-1 redox activity with APX3330, we observed reduced expression of G-CSF and the chemoattractant KC, followed by corrections in GMPs, CLPs, and MEPs in both the BM and spleen of cUC mice.

Circulating monocytes play a crucial role in maintaining colon tissue homeostasis. Pro-inflammatory Ly6C^hi^ monocytes are generated from common monocyte progenitor (cMoP) cells in the BM and released into circulations in a CCR2-dependent manner^38–40^. Consistent with these findings, cUC resulted in reduced Ly6C^hi^ monocytes in the BM and increased accumulation in the spleen, indicating enhanced emigration of pro-inflammatory Ly6C^hi^ monocytes to peripheral tissues, including the inflamed colon^41^. Notably, APX3330 treatment corrected the balance between Ly6C^hi^ and Ly6C^lo^ monocytes in the BM and spleen of cUC mice. This finding is of high translational relevance, as both patients with UC and murine models exhibited elevated circulating Ly6C^hi^ monocytes. In parallel, APX3330 treatment improved body weight loss, DAI scores, colon length, and normalized mature immune cell populations, including CD11b^+^Gr-1^+^ neutrophils, B220^+^ B cells, and CD3^+^ T cells in the colon, PB, BM, and spleen in cUC mice.

Mechanistically, we observed elevated serum TNF-α levels and activated NF-ĸB signaling in the BM of cUC mice, as evidenced by increased phosphorylation of IκBα, which is proposed to be a downstream target of APE1/Ref-1 signaling^33,66^. Additionally, we explored the activation of HIF-1α, a transcription factor that regulates cellular responses to hypoxia downstream of APE1/Ref-1^33^. Although HIF-1α is typically degraded under normoxic conditions, G-CSF stabilizes and activates HIF-1α even in the absence of hypoxia^45,67^. We hypothesized that G-CSF secreted during cUC activates HIF-1α in HSPCs and that APX3330 treatment inhibits this process. Consistent with this hypothesis, we observed elevated HIF-1α expression in primitive LK cells in the BM of cUC mice. HIF-1α activation in HSPCs likely promotes myeloid differentiation and mobilization of Ly6G^-^CD11b^+^Ly6C^hi^ monocytes to inflamed colon tissues, thereby exacerbating intestinal pathology^68^. Various studies have demonstrated that inflammation-induced stress and infection are known to influence HSC fate and function^18,56,69,70^. In agreement with these studies, we demonstrate that HSCs from cUC mice are functionally impaired in their engraftment capacity. Intriguingly, HSCs derived from cUC mice treated with APX3330 exhibited significantly improved stem cell functionality in competitive transplantation assays. Moreover, transplantation of HSCs from APX3330-treated cUC donors normalized colon pathology in recipient mice, indicating that APX3330 exerts stem-cell-autonomous effects.

To further elucidate downstream signaling mechanisms, we targeted HIF-1α with the specific inhibitor echinomycin. Pharmacological inhibition of HIF-1α phenocopies the effects of ref-1 inhibition by APX3330, restoring the HSC and HSPC pools in the BM and spleen, and reversing key features of cUC, including body weight loss, DAI score, colon length, and splenomegaly. These effects were accompanied by reduced expansion of IL-1r1-expressing HSPC populations, indicating a crucial role for IL-1r1 signaling in intestinal inflammation. IL-1r1 signaling is a well-established driver of inflammatory monocyte recruitment and intestinal inflammation, particularly through IL-1β-dependent pathways^55,57^. Consistent with prior studies showing impaired monocyte infiltration upon global loss of IL-1 signaling^56^, our data indicate that modulation of this pathway substantially alters myeloid cell dynamics during cUC. The restoration of the balance between pro-inflammatory Ly6C^hi^ and anti-inflammatory Ly6C^lo^ monocytes following echinomycin treatment parallels the effects observed with ref-1 inhibition by APX3330, suggesting that IL-1r1 signaling functions downstream of HIF-1α in cUC inflammatory context^71^. To directly test whether IL-1r1-signaling mediates inflammation-driven hematopoietic dysfunction in cUC, we employed *IL-1r1*-deficient donor cells in competitive transplantation assays. Importantly, genetic ablation of *IL-1r1* conferred partial but significant protection against DSS-induced chronic UC, as evidenced by attenuated myeloid expansion, preservation of colon length, and reduced accumulation of pathogenic Th17 cells in the colon. Together, these findings support a model in which HIF-1α promotes inflammatory signaling through the IL-1r1 axis, driving aberrant HSPC differentiation and sustained myelopoiesis under cUC conditions.

Collectively, our findings define an APE1/Ref-1/HIF-1α/IL-1r1 signaling axis that links chronic intestinal inflammation to systemic hematopoietic dysfunction. We propose a feed-forward model in which intestinal inflammation induces ref-1-dependent redox signaling, stabilizes HIF-1α, and amplifies IL-1r1-driven inflammation, thereby enforcing sustained myelopoiesis. Targeting APE1/Ref-1/HIF-1α/IL-1r1 signaling axis restores hematopoietic homeostasis and mitigates intestinal pathology, revealing hematopoietic reprogramming as a key driver of UC.

## Materials and methods

### Mice

C57BL/6 (B6) male mice were procured from the In Vivo Core facility at Indiana University. All mice were housed in pathogen-free standard laboratory conditions (22 ± 1 °C Temp, 12:12-h light/dark cycle) and given ad libitum access to a normal chow diet and water throughout the study at the Indiana University School of Medicine.

### Chronic DSS-induced ulcerative colitis model

Eight to ten-week-old B6 mice were used to develop a chronic ulcerative colitis (cUC) model by oral administration of DSS (MW 36–50 kDa colitis grade, MP Biomedicals, Santa Ana, CA) in sterile drinking water. We used 3.5% (w/v) DSS, which effectively induced cUC when mice were treated for 5 cycles consisting of 7 days of DSS administration followed by 5 days of rest (drinking water). The day DSS started was considered day 0. Body weight, stool softness, and blood in the rectum or stool were recorded daily during DSS and recovery cycles. One week after the final dose of DSS, mice were sacrificed, and tissue samples were harvested for analysis (Fig. S1a).

### Assessment of ulcerative colitis (UC) severity

To evaluate the progression of ulcerative colitis, a disease activity index (DAI) was assessed during the DSS treatment cycles as reported previously^72^. The DAI is an aggregate score derived from factors such as body weight loss, stool softness, and blood in the stool or rectum. Body weight loss was scored as follows: score 0, no weight loss compared to initial weight; score 1, weight loss within 1–5%; score 2, weight loss within 5–10%; score 3, weight loss within 10–20%; and score 4, weight loss >20%. Stool consistency score was determined as follows: score 0, normal (solid pellet); score 1, soft but in pellet shape; score 2, loose stool but with some solidity; score 3, loose stool with signs of liquid consistency; and score 4, watery diarrhea. Rectal bleeding was scored as follows: score 0, no sign of blood; score 1, no bleeding; score 2, slight bleeding; score 3, bloody diarrhea; and score 4, gross bleeding.

### Assessment of the progression of myelopoiesis

The progression of myelopoiesis in mice was assessed in the resting phase after each DSS treatment cycle by counting white blood cells (WBCs) using Element HT5-Heska and flow-cytometry analysis of peripheral blood stained with CD11b, Gr-1, and B220 antibodies.

### Colon length measurement

Colon lengths were measured on sacrifice day from the ileocecal junction to the rectum, and the unit was expressed in centimeters (cm).

### Isolation of lymphocytes from the lamina propria (LP) of the colon

Colons were opened longitudinally and washed gently with PBS. Then colons were excised into small pieces and incubated at 37 °C in RPMI 1640 (Lonza) supplemented with 5% heat-inactivated fetal bovine serum (FBS), 100 U/mL penicillin, 100 mg/mL streptomycin, 25 nmol/L HEPES, 2mM L-glutamate, 55 mM 2-ME, and 2 mM EDTA (to remove epithelial cells). After three 20-min washes to remove ETDA, the tissue was transferred to complete RPMI 1640 containing 1 mg/mL Collagenase IV (Sigma) and 200 U/mL DNase I (Sigma) and incubated at 37 °C for 1 h. The suspension was passed through a 70 µM nylon filter and then centrifuged at 450 × *g* for 10 min at 4 °C. The cells were then pelleted and washed once more with ice-cold complete RPMI 1640 to remove collagenase. LP lymphocytes were enriched on a 40/70% Percoll (GE Healthcare) gradient centrifugation for 20 min at 750 × *g* speed at 21 °C without acceleration and brake. Cells were then collected for downstream analyses.

### Immunophenotyping

Immunophenotyping of cells was executed as described before^73^. A single cell suspension of bone marrow and spleen was prepared. Briefly, bone marrows were flushed with Iscove Modified Dulbecco Medium (IMDM, Invitrogen). To prepare single-cell splenocytes, spleens were crushed between microscopic slides, flushed with IMDM using a syringe, and filtered through a 50 μm nylon filter. Red blood cells (RBCs) from bone marrow and splenocytes were lysed in a 0.8% NH4Cl RBC lysis buffer for 5 min at room temperature, and then cells were resuspended in phosphate-buffered saline containing 1% bovine serum albumin (BSA, Sigma) and 10% rat serum (Sigma). After counting the cells in the cell viability analyzer (Vi-CELL-XR, Bechmann Coulter), an equal number of cells was used for staining with flow antibodies. Intracellular flow cytometry (ICFC) was performed as per^74^. Briefly, freshly prepared bone marrow cells and colonic LPL cells were pre-stained by using cell surface antibodies and then fixed with BioLegend Cytofix and washed with BioLegend Cytoperm three times. Then, pre-stained cells were stained with the indicated antibodies against intracellular proteins. Staining with an Annexin-V and 7-AAD kit (BioLegend, Cat # 640922) was performed according to the manufacturer’s instructions for apoptosis analysis. The stained cells were subjected to multi-parametric flow cytometric analysis using FASC Canto, LSR-II, and LSR Fortessa flow cytometers with Diva software (BD Biosciences), and the data were analyzed using FlowJo software (v10.7.0)^53^. A list of flow-antibodies is provided in supplementary Table S1.

### Serum cytokine profiling

Serum was separated from peripheral blood and submitted to Eve Technologies (Canada) for serum cytokine analysis^53^. Results are expressed as pg/mL or as ng/mL of serum.

### Competitive repopulation assay

For the competitive bone marrow transplantation (cBMT) assay, recipient (F1 mice, CD45.1^+^/CD45.2^+^) mice were irradiated at 1100 cGy from a cesium source using a split two dose of 700 and 400 cGy at an interval of 4 h. 0.2 × 10^6^ BM mono-nuclear donor cells from either B6-control or B6-mice treated with 3.5% DSS (w/v) (CD45.2^+^), along with 0.2 × 10^6^ Boy/J (CD45.1^+^) BM mono-nuclear competitor cells, were transplanted by intravenous injection into lethally irradiated F1 recipient mice (*n* = 5 per group). Engraftment of donor cells was determined at an interval of 4 weeks by flow cytometry analysis of peripheral blood. At 16 weeks post-transplantation, mice were euthanized for complete analysis (a schematic is shown in Fig. S5B). In another cBMT assay, where 0.2 × 10^6^ BM mono-nuclear donor CD45.2+ cells from either veh, DSS + veh (cUC), or DSS + APX3330 (cUC + APX3330) treated B6-mice were used along with 0.2 × 10^6^ Boy/J (CD45.1^+^) BM mono-nuclear competitor cells, which were transplanted by intravenous injection into lethally irradiated F1 recipient mice (*n* = 5 per group). Engraftment of donor cells was determined as above, and mice were sacrificed for complete analysis at the 20^th^ week post-transplantation (Fig. 4a).

### In vivo APE1/Ref-1 inhibitor APX3330 and HIF-1α-specific inhibitor Echinomycin drug treatment

For APX3330 treatment, mice were randomized into the following groups: vehicle (veh) control, DSS+veh, and APX3330 + DSS group (*n* = 5 mice per group). APX3330 drug, a kind gift from Dr. Mark R. Kelley at the Department of Pediatrics, Indiana University School of Medicine, Indianapolis, was dissolved in Cremophor: Ethanol (1:1) (Cremophor from Sigma, catalog no. C5135) for stock solution preparation and diluted with 0.5% methylcellulose prior to use. Mice were treated either with vehicle or APX3330 drug at 50 mg/kg body weight, twice a day by oral gavage, one week before the start of DSS treatment, and continued throughout the cycles of DSS treatment. For echinomycin (MedChemExpress catalog #HY-106101) treatment, mice were treated either with DMSO-vehicle or echinomycin at 10 µg/kg body weight on alternate days through intraperitoneal (i.p.) injection. The disease progression was monitored as described above. One week after the final dose of DSS, mice were sacrificed, and tissues were harvested for analysis.

### Statistical analysis

All data were presented as mean ± SEM. Statistical significance between groups was analyzed using GraphPad Prism software version 7.0 (GraphPad, San Diego, CA). For the analyses, the statistical significance was assessed by either one-way analysis of variance (ANOVA) with Tukey’s multiple comparison test, two-way ANOVA with Sidak’s multiple comparison test, or an unpaired Student’s two-tailed *t* test. *P* values < 0.05 were considered statistically significant.

### Study approval

All mouse studies were approved by the Indiana University Laboratory Animal Resource Center (Indianapolis, Indiana, USA), and all animal procedures were conducted in accordance with the IACUC at Indiana University School of Medicine.

### Reporting summary

Further information on research design is available in the Nature Portfolio Reporting Summary linked to this article.

## Supplementary information

Supplementary Information

Description of Additional Supplementary Materials

Supplementary Data 1

Reporting Summary

## Data Availability

All data generated or analyzed during this study are included in this published article and its Supplementary Material files. The numerical source data for all graphs in the manuscript can be found in “Supplementary Data 1” file.
