# Supplementary material for: Ref-1 drives ulcerative colitis induced systemic defects in hematopoietic cells
Source: Commun Biol. 2026 Mar 19;9:635. doi: 10.1038/s42003-026-09860-z (PMC13168444; doi:10.1038/s42003-026-09860-z)
Supplement: Supplementary file 1 — Supplementary Information [file 42003_2026_9860_MOESM1_ESM.pdf]

Supplementary Materials for

Ref-1 Drives Ulcerative Colitis induced Systemic Defects in Hematopoietic Cells

Ramesh Kumar *et al.*

Corresponding author: Reuben Kapur, rkapur@iu.edu.

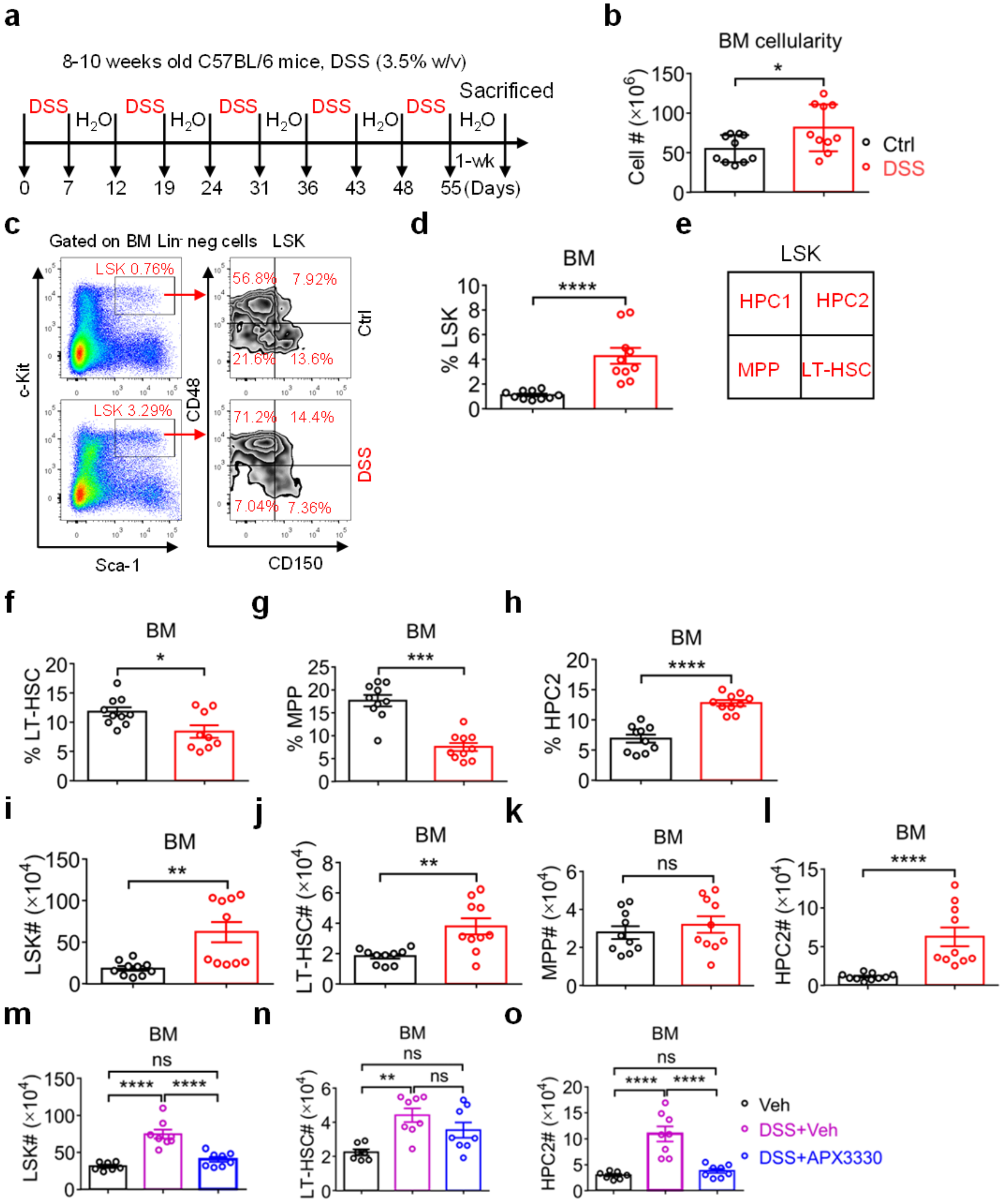

a
8-10 weeks old C57BL/6 mice, DSS (3.5% w/v)
DSS
H2O
Sacrificed
1-wk
0 7 12 19 24 31 36 43 48 55 (Days)
b
BM cellularity
Cell # (×10^6)
Ctrl
DSS
c
Gated on BM Lin neg cells
LSK
LSK 0.76%
LSK 3.29%
56.8% 7.92%
21.6% 13.6%
71.2% 14.4%
7.04% 7.36%
c-Kit
Sca-1
CD48
CD150
Ctrl
DSS
d
BM
% LSK
e
LSK
HPC1
HPC2
MPP
LT-HSC
f
g
h
i
j
k
l
m
n
o
% LT-HSC
% MPP
% HPC2
LSK# (×10^4)
LT-HSC# (×10^4)
MPP# (×10^4)
HPC2# (×10^4)
ns
Veh
DSS+Veh
DSS+APX3330

fig. S1. DSS-induced chronic ulcerative colitis (cUC) reduces frequency of LT-HSCs, MPPs (ST-HSCs) and enhances restricted myeloid-biased (HPC-2) in the BM.

(a) Experimental scheme illustrating the cycles of DSS treatment. 8-10 weeks old C57BL/6 mice were treated with 3.5% DSS (w/v) in sterile water for 7 days followed by a resting phase for 5 days, and this treatment was repeated 5 times. Control mice received sterile water. **(b)** The total bone marrow (BM) cells in cUC and control mice. **(c)** Representative flow cytometry profiles showing gating of BM Lineage $(Lin)^-$, Sca-1$(S)^+$, c-Kit $(K)^+$ (LSK cells, left panel) and characterization of hematopoietic stem cell subsets gated on LSK cells (right panel). **(d)** Shows the frequency of LSK cells in the BM of cUC and control mice**.** **(e)** Gating strategy for hematopoietic stem cell subsets in LSK population. Frequencies of LT-HSCs, LSK^{gated} $CD150^+CD48^-$ **(f)**, MPPs, LSK^{gated} $CD150^-CD48^-$ **(g)**, and HPC2, LSK^{gated} $CD150^+CD48^+$ **(h)** within the LSK cells in the BM of cUC and control mice. **(i)** The absolute number of LSKs in the BM of cUC and control mice. The absolute numbers of **(j)** LT-HSCs, **(k)** MPPs and **(l)** HPC2 within the LSK cells in the BM of cUC and control mice. The absolute numbers of LSKs **(m),** LT-HSCs **(n)** and HPC2 **(o)** in the BM of Veh, DSS+Veh (cUC) and DSS+APX3330 (cUC+APX3330) treated mice. Results are the cumulative data of two independent experiments (**b, d, f-l;** n=10 mice per group**; m-o;** n=7-8 mice per group), and each dot represents an individual mouse. Data are shown as mean ± SEM. Statistical significance was determined using either an unpaired Student's two-tailed T-test (**b, d, f-l**) or one-way ANOVA with Tukey's multiple comparison test **(m-o**) for the for the analysis of differences between the experimental groups. $^{*}P<0.05$, $^{**}P<0.01$, $^{***}P<0.001$, and $^{****}P<0.0001$; n.s., not significant.

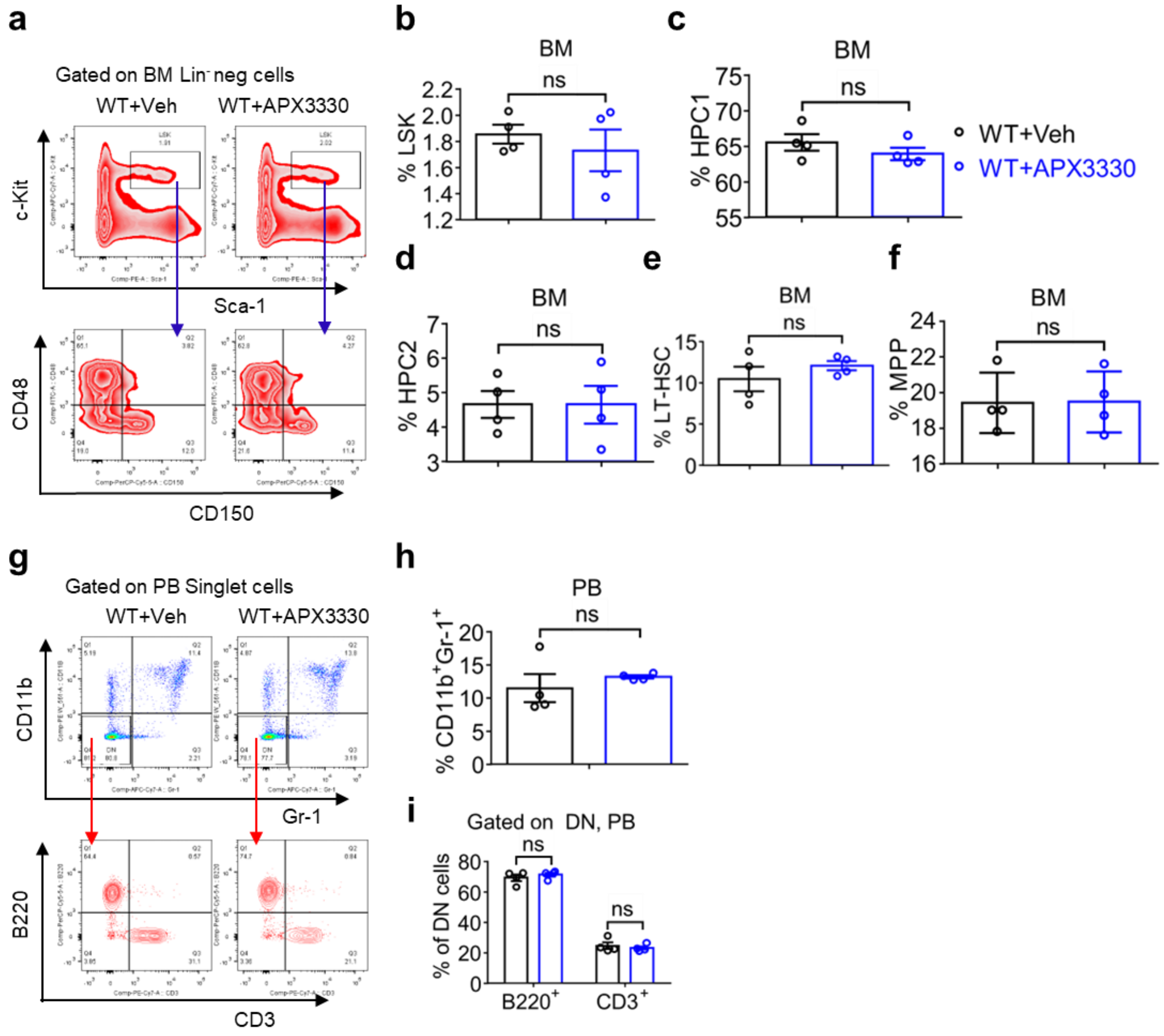

fig. S2. APX3330 treatment does not affect normal hematopoiesis.

(a) Representative flow cytometry profiles showing gating of BM Lineage (Lin)$^{-}$, Sca-1(S)$^{+}$, c-Kit (K)$^{+}$ (LSK cells, top panel) and characterization of hematopoietic stem cell subsets gated on LSK cells (bottom panel). Frequencies of LSKs **(b)**, HPC1 cells **(c),** HPC2 cells **(d),** LT-HSCs **(e),** and MPPs **(f)** in the BM of WT control mice either treated with Veh or APX3330 alone**. (g)** Representative flow cytometry profiles showing gating of myeloid (top panel) and lymphocytes (bottom panel gated on double negative, CD11b^{-}Gr-1^{-}) on PB singlet cells. Frequencies of CD11b^{+}Gr-1^{+} neutrophils **(h)** and lymphocytes (B220^{+} B cells and CD3^{+} T cells) **(i)** in the PB of WT control mice either treated with Veh or APX3330 alone**.** Results are from 4 mice in each experimental group. Data are presented as mean ± SEM. Statistical significance was obtained

using either an unpaired, two-tailed Student's T-test **(b-f, h)** or two-way ANOVA **(i)** with Tukey's multiple comparison test for the analysis of differences between the experimental groups. $^{*}P<0.05$, $^{**}P<0.01$, $^{***}P<0.001$ and $^{****}P<0.0001$; n.s., not significant.

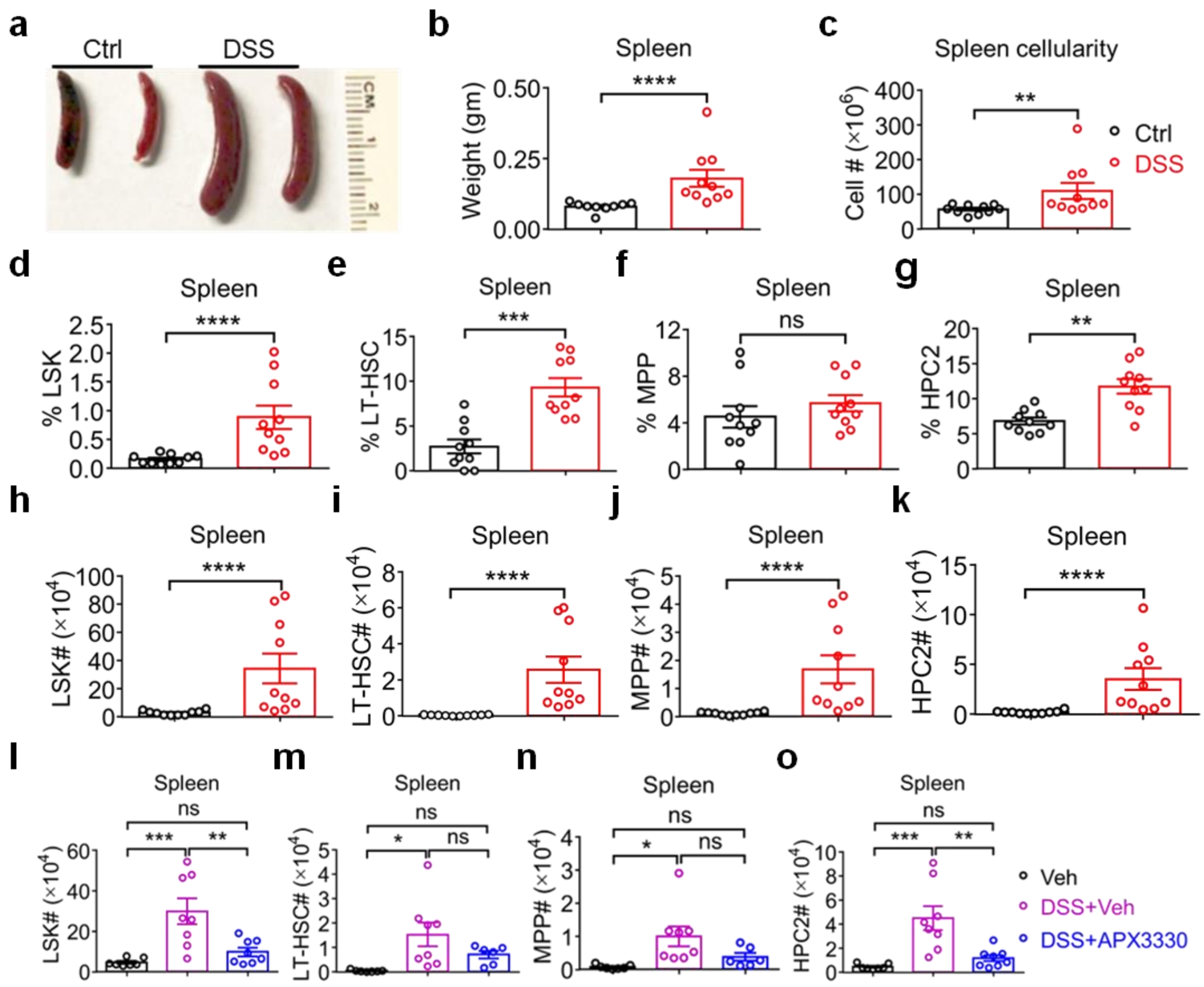

fig. S3. cUC leads to splenomegaly and "extramedullary hematopoiesis" (EMH) in the spleen.

(a) Representative graph of the spleen from cUC and control mice. (**b)** Shows spleen weight in grams, and **(c)** shows spleen cellularity in cUC and control mice. **(d)** Shows the frequency of LSK cells in the spleen of cUC and control mice. Frequencies of LT-HSCs **(e)**, MPPs **(f)**, and HPC2 **(g)** within the LSK cells in the spleen of cUC and control mice**. (h)** The absolute number of LSKs in the spleen of cUC and control mice. The absolute numbers of LT-HSCs **(i)**, MPPs **(j)** and HPC2 **(k)** in the spleen of cUC and control mice. The absolute numbers of LSKs **(l),** LT-HSCs **(m),** MPPs **(n),** and HPC2 **(o)** in the spleen of Veh, DSS+Veh (cUC) and DSS+APX3330 (cUC+APX3330) treated mice. Results are the cumulative data of two independent experiments (**b-k;** n=10 mice

per group**; l-o;** n=7-8 mice per group), and each dot represents an individual mouse. Data are shown as mean ± SEM. Statistical significance was determined using either an unpaired Student's two-tailed T-test (**b-k**) or one-way ANOVA with Tukey's multiple comparison test **(l-o**) for the analysis of differences between the experimental groups. $^{*}P<0.05$, $^{**}P<0.01$, $^{***}P<0.001$, and $^{****}P<0.0001$; n.s., not significant.

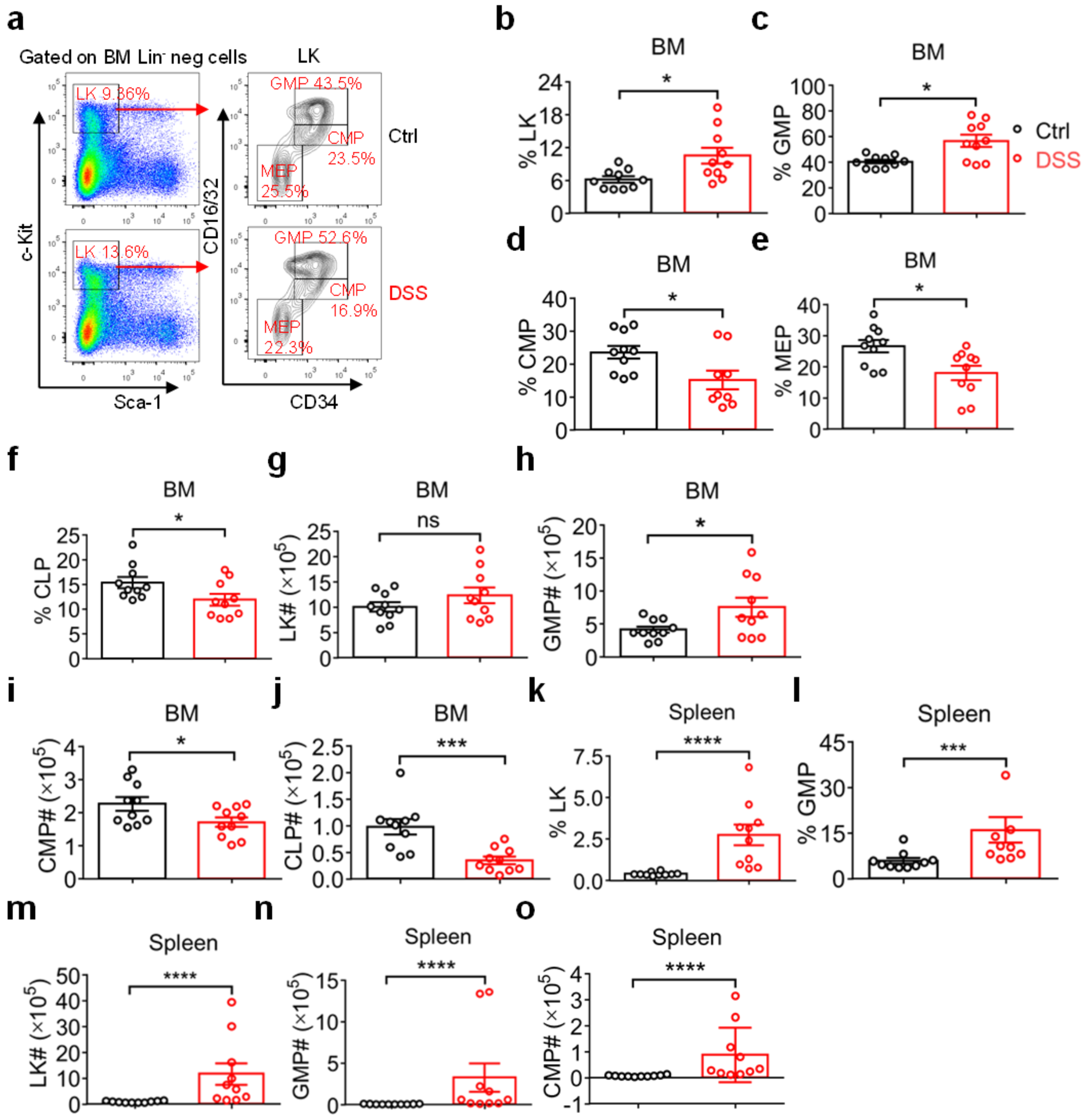

fig. S4. cUC drives hematopoiesis toward myelopoiesis in the BM and spleen.

(**a**) Shows flow-cytometric analysis of BM immature Lin$^-$, c-Kit$^+$ Sca-1$^-$ (LK cells, left panel) and hematopoietic stem and progenitor cells (HSPC) subsets gated on LK cells (right panel). **(b)** Shows the frequency of immature LK cells in the BM of cUC and control mice. Frequencies of **(c)** granulocyte-macrophage progenitors GMPs, LK^{gated}CD16/32$^+$CD34$^+$, **(d)** common-myeloid progenitors CMPs, LK^{gated}CD16/32^{lo}CD34$^+$, **(e)** megakaryocyte-erythroid progenitors MEPs, LK^{gated}CD16/32$^-$CD34$^-$ within the LK cells in the BM of cUC and control mice. **(f)** Shows the

frequency of common-lymphoid progenitors CLP, $Lin^{-}CD127^{+}c\text{-}Kit^{lo}Sca\text{-}1^{lo}$ in the BM of cUC and control mice. The absolute numbers of **(g)** LKs, (**h)** GMPs, **(i)** CMPs, and **(j)** CLPs in the BM of cUC and control mice. **(k)** Shows the frequency of LKs, and **(l)** shows the frequency of GMPs within the LK cells in the spleen of cUC and control mice. The absolute numbers of **(m)** LKs, **(n)** GMPs, and **(o)** CMPs in the spleen of cUC and control mice. Results are pooled data of two independent experiments (n=10 mice per group), and each dot represents an individual mouse. Data are presented as mean ± SEM. Statistical significance was obtained using an unpaired, two-tailed Student's T-test for the analysis of differences between the experimental groups. $^{*}P<0.05$, $^{***}P<0.001$ and $^{****}P<0.0001$; n.s., not significant.

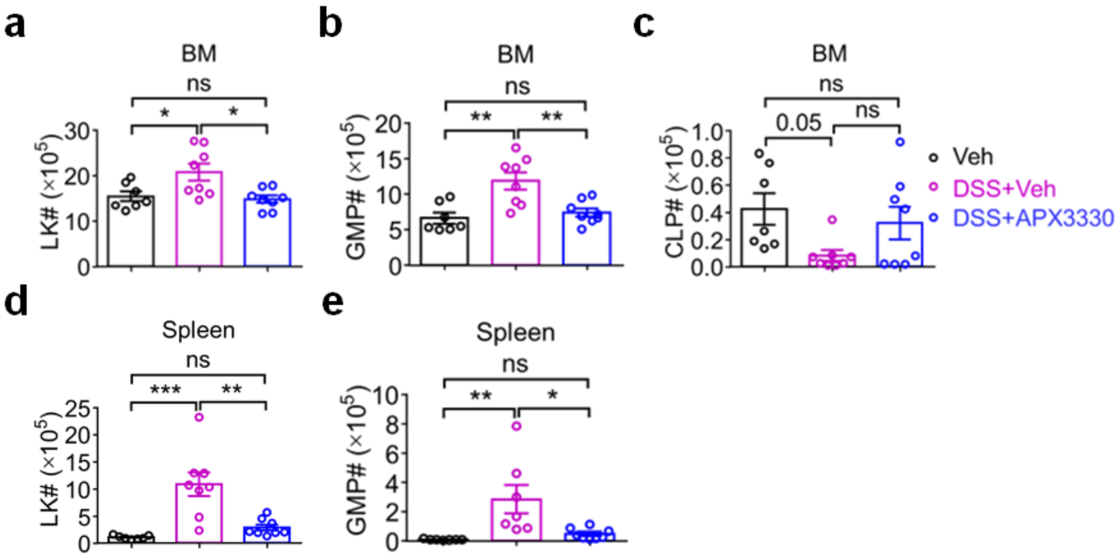

fig. S5. APX3330 treatment normalizes cUC-induced changes in the absolute number of HSPC subsets in the BM and spleen.

The absolute numbers of LK cells **(a)**, GMPs **(b)**, and CLPs **(c)** in the BM of Veh, DSS+Veh (cUC) and DSS+APX3330 (cUC+APX3330) treated mice. The absolute numbers of LKs **(d)**, and GMPs **(e)** in the spleen of Veh, DSS+Veh (cUC) and DSS+APX3330 (cUC+APX3330) treated mice. Results are pooled data of two independent experiments, and each dot represents an individual mouse (n=7-8 mice per group). Data are presented as mean ± SEM. Statistical significance was obtained using one-way ANOVA with Tukey's multiple comparison test for the analysis of differences between the experimental groups. $^{*}P<0.05$, $^{**}P<0.01$, and $^{***}P<0.001$; n.s., not significant.

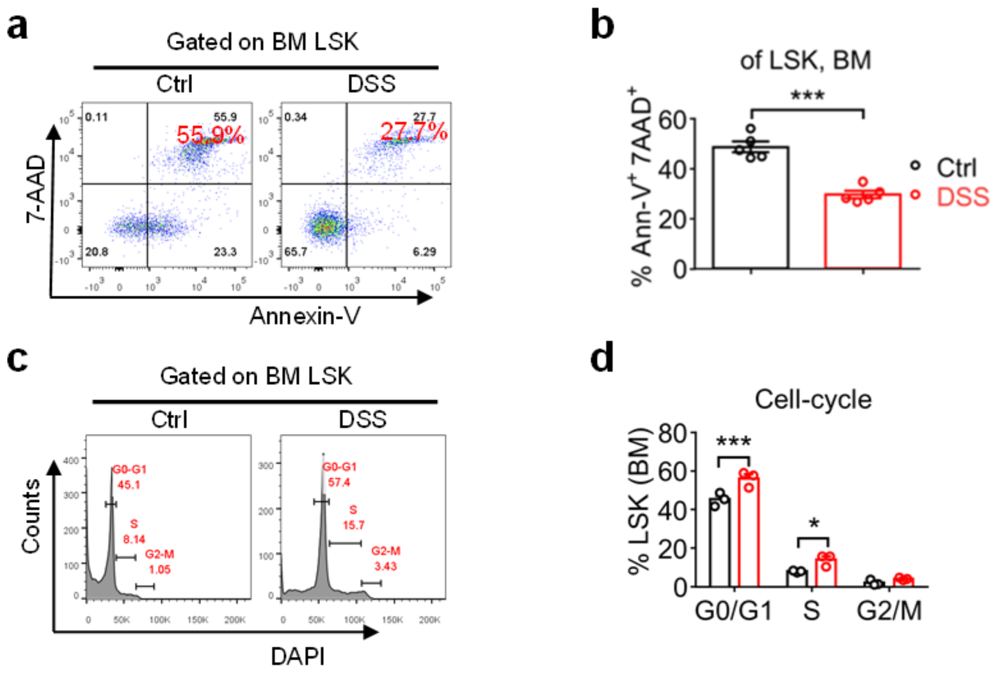

fig. S6. cUC causes reduced apoptosis and increased accumulation of BM LSK cells in G0/G1 and S phase of cell-cycle.

(a) Represents the flow-cytometric analysis of apoptosis in BM LSK cells stained with 7-AAD and Annexin-V from cUC and control mice. **(b)** Shows the frequency of BM LSK apoptotic (7-AAD$^+$Annexin-V$^+$) cells in cUC and control mice. **(c)** Representative flow-plot showing analysis of cell cycle of BM LSK cells using DAPI staining. **(d)** Quantification of BM LSK cells in G_0/G1, S and G2/M phase. Results are from a single experiment (b**;** n=5 and d**;** n=3 mice per group) and each dot represents an individual mouse. Data are presented as mean ± SEM. Statistical significance was determined using either an unpaired Student's two-tailed T-test **(b)** or two-way ANOVA with Sidak's multiple comparison test **(d)** for the analysis of differences between the experimental groups. $^{*}P$<0.05, and $^{***}P$<0.001.

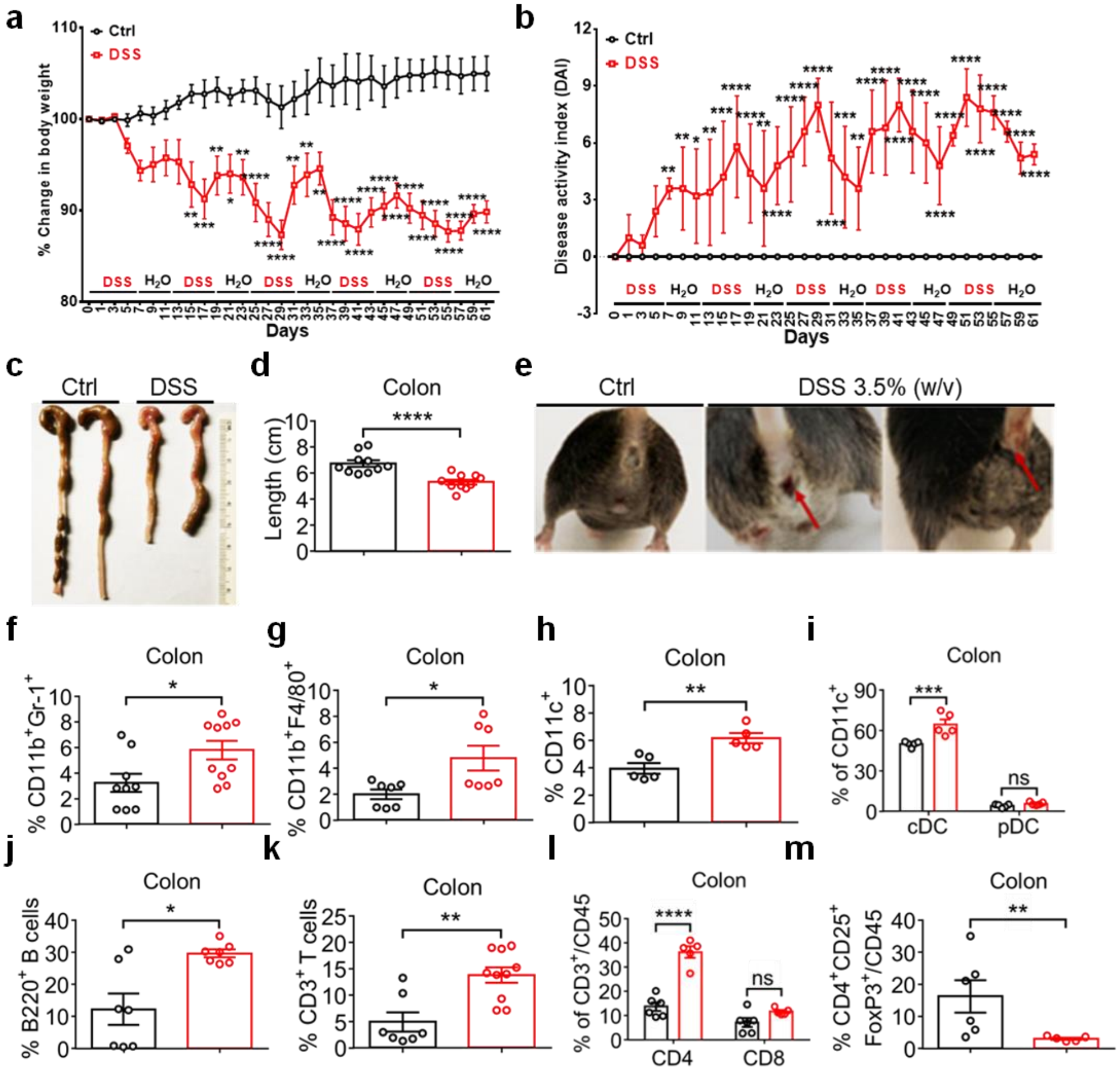

fig. S7. Mice treated with DSS show key features of UC and an altered immune response in the colon.

(a) Shows the changes in body weight and **(b)** disease activity index (DAI) in cUC and control mice. **(c)** Representative graphs showing colon tissue, and **(d)** quantification of colon length expressed in centimeters (cm) in cUC and control mice. **(e)** Mice showing diarrhea and rectal bleeding indicated by red arrow. Frequencies of $CD11b^{+}Gr\text{-}1^{+}$ neutrophils **(f)**, $CD11b^{+}F4/80^{+}$ macrophages **(g)**, $CD11c^{+}$ myeloid cells **(h)**, c-DC ($CD11c^{+}CD11b^{+}B220^{-}$) and p-DC ($CD11c^{+}B220^{+}CD11b^{-}$) **(i)**, $B220^{+}$ B cells **(j)** and $CD3^{+}$ T cells **(k)**, $CD4^{+}$ and $CD8^{+}$ T cells **(l)**, T reg

cells **(m)**, within the $CD45^+$ leukocytes in the colon of cUC and control mice. Results are either representative data of two independent experiments (**a-b;** n=5 mice per group) or cumulative data of two independent experiments (**d & f-m;** n=5-10 mice per group), and each dot represents an individual mouse. Data are presented as mean ± SEM. Statistical significance was determined using either two-way ANOVA with Sidak's multiple comparison test **(a-b, and i & l)** or an unpaired Student's two-tailed T-test **(d, f-h, and j-k & m)** for the analysis of differences between the experimental groups. $^{*}P<0.05$, $^{**}P<0.01$, $^{***}P<0.001$, and $^{****}P<0.0001$; n.s., not significant.

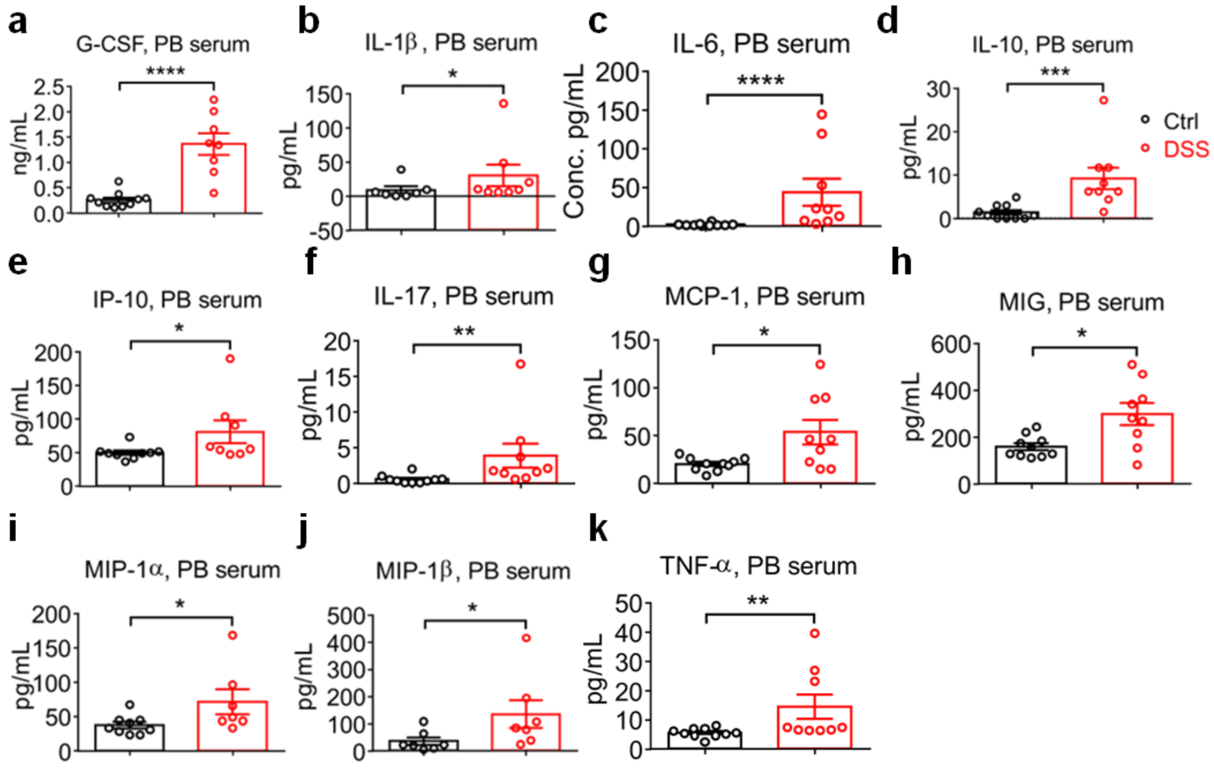

fig. S8. cUC causes altered cytokine/chemokine expression in the peripheral blood (PB) serum.

Multiplex serum profiling for cytokine and chemokine in DSS treated and control mice. The quantitative data show cytokine and chemokine concentrations expressed in either ng/mL or pg/mL for **(a)** G-CSF, **(b)** IL- 1β, **(c)** IL-6, **(d)** IL-10, **(e)** IP-10, **(f)** IL-17, **(g)** MCP-1, **(h)** MIG, **(i)** MIP-1α, **(j)** MIP-1β, and **(k)** TNF-α in PB serum of DSS treated and control mice. Results are pooled data of two independent experiments (n=8-10 mice per group), and each dot represents an individual mouse. Data are presented as mean ± SEM. Statistical analysis was performed using an unpaired two-tailed Student's T-test for the analysis of differences between the experimental groups. $^{*}P<0.05$, $^{**}P<0.01$, $^{***}P<0.001$, and $^{****}P<0.0001$.

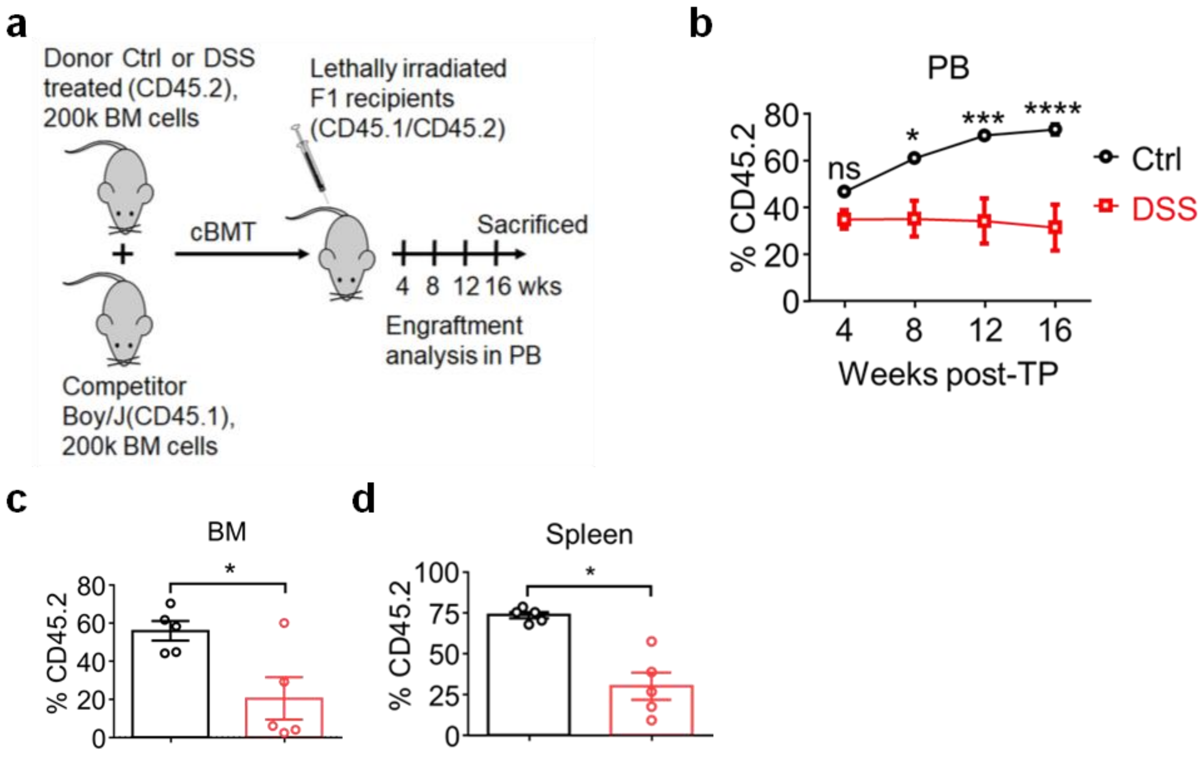

fig. S9. cUC causes reduced engraftment ability of HSCs in a transplant setting.

(a) A schematic presentation of primary competitive bone-marrow transplantation (cBMT) assay.

(b) Analysis of PB engraftment at 4, 8, 12, and 16 weeks post-cBMT in F1 recipient mice. The analyses of engraftment of donor-derived cells: **(c)** in the BM (% $CD45.2^{+}$ cells) and **(d)** in the spleen (% $CD45.2^{+}$ cells) of F1 recipient mice. Results are from 4-5 mice per group. Data are presented as mean ± SEM. Statistical analysis was performed using either two-way ANOVA **(b)** with Sidak's multiple comparison test or an unpaired, two-tailed Student's T-test **(c-d)** for the analysis of differences between the experimental groups. $^{*}P<0.05$, $^{***}P<0.001$, and $^{****}P<0.0001$; n.s., not significant.

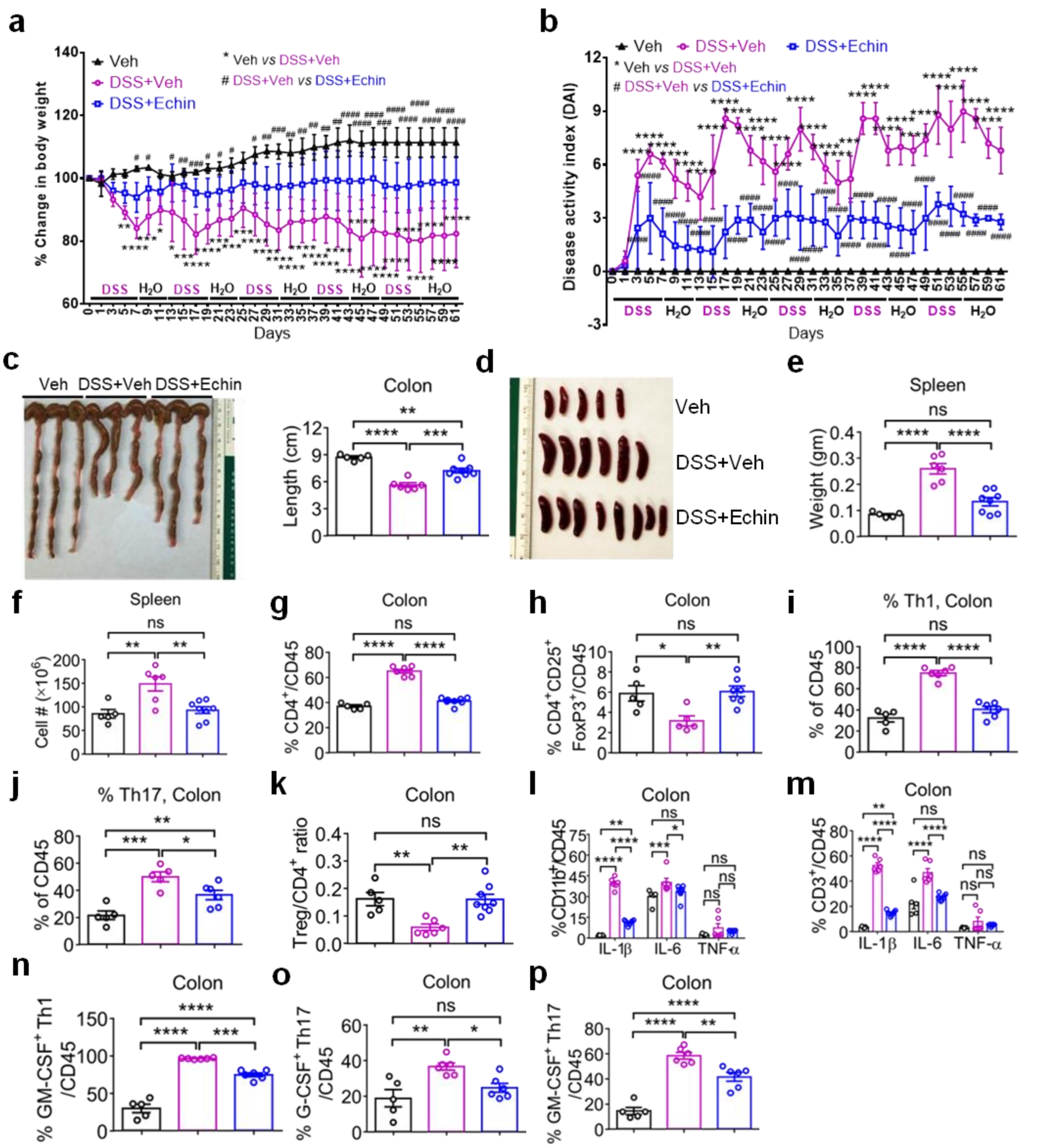

fig. S10. HIF-1α inhibitor, echinomycin treatment reverses cUC-induced changes in mice. Mice were treated with echinomycin at 10 μg/Kg, mouse body weight, on alternate days through intraperitoneal injection. The treatment regimen started 7 days before the initiation of DSS treatment and continued throughout the cycles of DSS treatment. **(a)** Changes in body weight, and **(b)** DAI during DSS treatment. **(c)** Representative graphs for colon tissue from Veh, DSS+Veh

(cUC), and DSS+Echin (cUC+Echin) treated mice, and quantification of colon length expressed in centimeters (cm). **(d)** Representative graphs for spleen, **(e)** spleen weight expressed in grams (gms), and **(f)** the total number of splenocytes in Veh, DSS+Veh (cUC), and DSS+Echin (cUC+Echin) treated mice. Frequencies of **(g)** $CD4^+$ T cells, **(h)** $CD4^+$ Treg cells, **(i)** Th1 cells, **(j)** Th17 cells, **(k)** ratio of Treg/$CD4^+$ T cells within the $CD45^+$ leukocytes in the colon of Veh, DSS+Veh (cUC), and DSS+Echin (cUC+Echin) treated mice. **(l)** Percentage of $CD11b^+$ myeloid cells, and **(m)** $CD3^+$ T cells expressing IL-1β, IL-6, and TNF-α cytokines within the $CD45^+$ leukocytes in the colon of Veh, DSS+Veh (cUC), and DSS+Echin (cUC+Echin) treated mice. **(n)** Percentage of GM-CSF^+ Th1 cells, **(o)** percentage of G-CSF^+ Th17 cells, and **(p)** GM-CSF^+ Th17 cells within $CD45^+$ leukocytes in the colon of Veh, DSS+Veh (cUC), and DSS+Echin (cUC+Echin) treated mice. Results are from a single experiment with mice (n=4 to 8) in each experimental group. Each dot represents an individual mouse. Data are shown as mean ± SEM. Statistical significance was determined using either two-way ANOVA **(a-b, l-m)** or one-way ANOVA **(c, e-k & n-p)** with Tukey's multiple comparison test for the analysis of differences between the experimental groups. $^{*}P<0.05$, $^{**}P<0.01$, $^{***}P<0.001$, and $^{****}P<0.0001$; n.s., not significant.

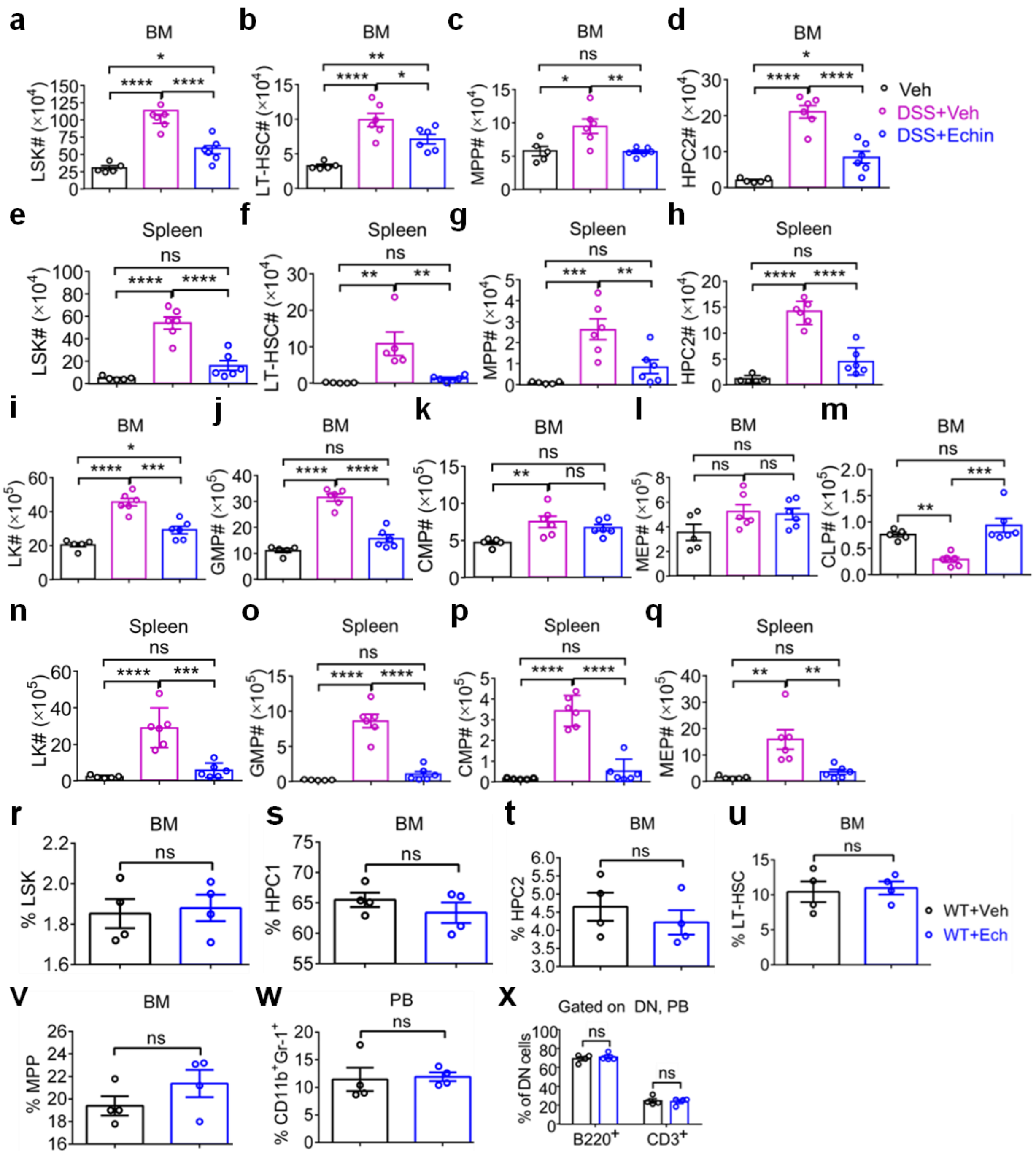

a BM LSK# (×10^4)
b BM LT-HSC# (×10^4)
c BM MPP# (×10^4)
d BM HPC2# (×10^4)
Veh
DSS+Veh
DSS+Echin
e Spleen LSK# (×10^4)
f Spleen LT-HSC# (×10^4)
g Spleen MPP# (×10^4)
h Spleen HPC2# (×10^4)
i BM LK# (×10^5)
j BM GMP# (×10^5)
k BM CMP# (×10^5)
l BM MEP# (×10^5)
m BM CLP# (×10^5)
n Spleen LK# (×10^5)
o Spleen GMP# (×10^5)
p Spleen CMP# (×10^5)
q Spleen MEP# (×10^5)
r BM % LSK
s BM % HPC1
t BM % HPC2
u BM % LT-HSC
WT+Veh
WT+Ech
v BM % MPP
w PB % CD11b+Gr-1+
x Gated on DN, PB
% of DN cells
B220+
CD3+

fig. S11. HIF-1α inhibitor, echinomycin normalizes cUC-induced hematopoietic defects by balancing myelopoiesis and lymphopoiesis in the BM and spleen.

The absolute numbers of **(a)** LSKs, **(b)** LT-HSCs, **(c)** MPPs, and **(d)** HPC2 in the BM, and **(e)** LSKs, (**f)** LT-HSCs **(g)** MPPs, and **(h)** HPC2 in the spleen of Veh, DSS+Veh (cUC), and DSS+Echin (cUC+Echin) treated mice. The absolute numbers of **(i)** LKs, **(j)** GMPs, **(k)** CMPs, **(l)** MEPs, and **(m)** CLPs in the BM, and **(n)** LKs, **(o)** GMPs, **(p)** CMPs, and **(q)** MEPs in the spleen of Veh, DSS+Veh (cUC), and DSS+Echin (cUC+Echin) treated mice. Frequencies of **(r)** LSKs, **(s)** HPC1 cells, **(t)** HPC2 cells, **(u)** LT-HSCs, and **(v)** MPPs in the BM of WT control mice either treated with Veh or echinomycin alone**.** Frequencies of **(w)** $CD11b^{+}Gr\text{-}1^{+}$ neutrophils, and **(x)** lymphocytes ($B220^{+}$ B cells and $CD3^{+}$ T cells) gated on double negative for CD11b/Gr-1 ($CD11b^{-}Gr\text{-}1^{-}$) cells in PB of WT control mice either treated with Veh or echinomycin alone**.** Results are from a single experiment with mice (n=4-8) in each experimental group. Each dot represents an individual mouse. Data are shown as mean ± SEM. Statistical significance was determined using one-way ANOVA with Tukey's multiple comparison test **(a-q)**, an unpaired, two-tailed Student's T-test **(r-w)** or two-way ANOVA **(x)** with Tukey's multiple comparison test for the analysis of differences between the experimental groups. $^{*}P<0.05$, $^{**}P<0.01$, $^{***}P<0.001$, and $^{****}P<0.0001$; n.s., not significant.

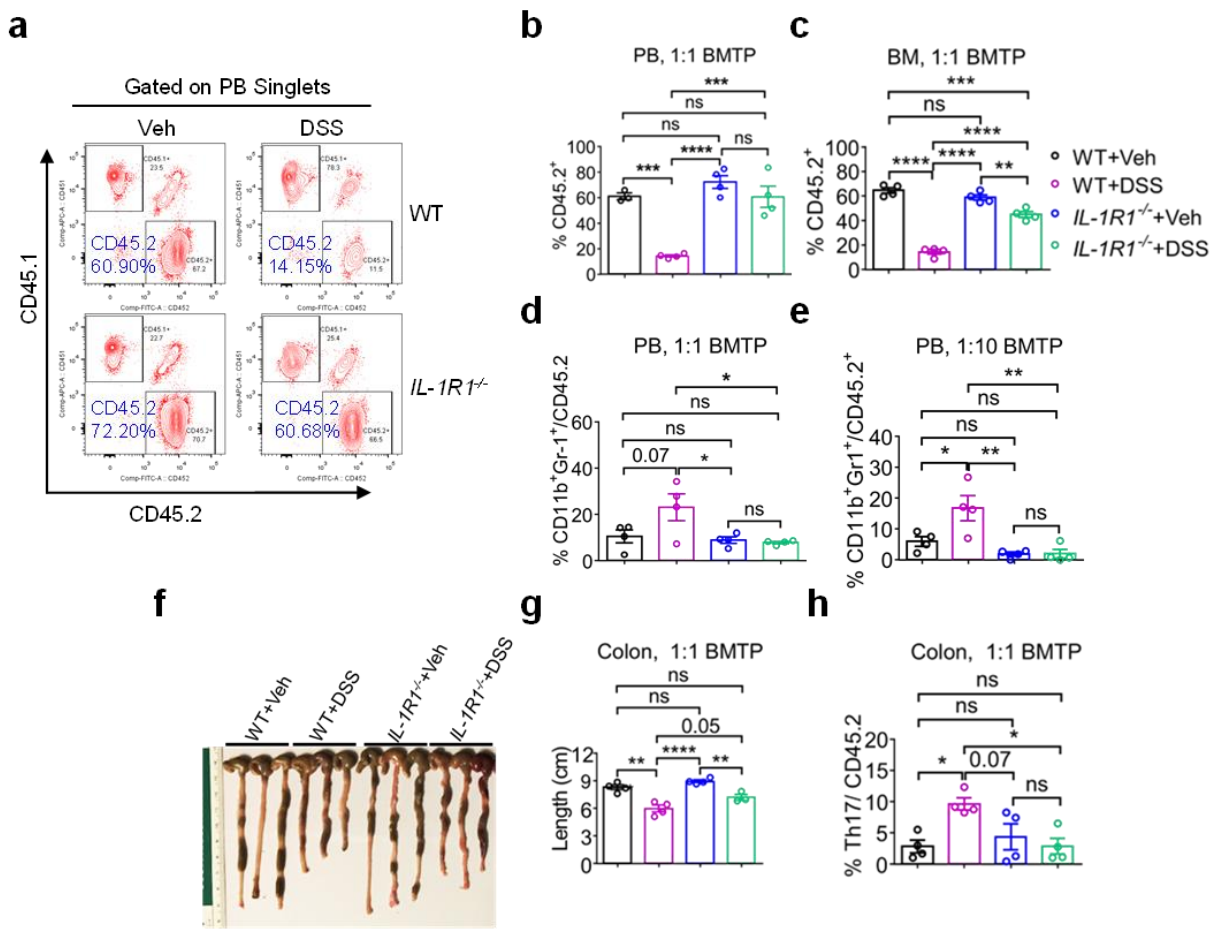

fig. S12. *IL-1r1* deficiency protects mice from developing cUC by reducing myeloid cells and pathogenic Th17 cells in the colon of mice in a transplant setting.

Lethally irradiated F1 recipient mice transplanted with CD45.2⁺ BM cells of WT control or *IL-1r1* KO mice mixed with CD45.1⁺ BM cells of Boy/J mice into 1:1 or 1:10 ratio. After 4th week post engraftment, F1 mice were either treated with Veh or with 3.5% DSS for 5-cycles. **(a**) Represents flow-gating strategy for donor-derived $CD45.2^+/CD45.1^+$ cells in PB of F1 recipient mice at 5th-cycle of DSS treatment. The frequencies of donor-derived $CD45.2^+$ cells in PB **(b)** and in the BM **(c)** of F1 recipient mice at 5th-cycle of DSS treatment. The frequency of donor- derived neutrophils ($CD11b^+Gr1^+$) in the PB **(d-e)** of F1 recipient at 5th-cycle of DSS treatment. **(f)** Shows images of colon tissues, and **(g)** shows the quantification of colon size in F1 recipient mice at 5th-cycle of DSS treatment. **(h)** The frequency of donor- derived Th17 cells ($CD4^+IL\text{-}17A^+/CD45^+$) in the colons of

F1 recipient mice at 5th-cycle of DSS treatment. Results are from 4 mice in each experimental group. Data are presented as mean ± SEM. Statistical significance was determined using one-way ANOVA with Tukey's multiple comparison test for the analysis of differences between the experimental groups. $^{*}P<0.05$, $^{**}P<0.01$, $^{***}P<0.001$, and $^{****}P<0.0001$; n.s., not significant.

Supplementary Table S1: List of reagents used in current study.

Reagents	Company	Catlog No.
Mouse Flow Antibodies		
Lineage cocktail antibodies Pacific Blue	Biolegend	Cat # 133310
C-KIT APC	Biolegend	Cat # 105812
Sca1 PE	Biolegend	Cat # 108108
CD48 FITC	Biolegend	Cat # 103404
CD150 PerCPCy5.5	Biolegend	Cat # 115922
CD34 FITC	eBiosciences	Cat #11-0341-82
CD16/32 PE Cy7	Biolegend	Cat # 101318
C-KIT APC Cy7	Biolegend	Cat # 105826
CD127 BV605	Biolegend	Cat # 135041
CD45 Pacific Blue	Biolegend	Cat # 157212
B220 PE	Biolegend	Cat # 103208
B220 PerCPCy5.5	Biolegend	Cat # 103236
Gr1 PerCPCy5.5	Biolegend	Cat # 108408
Gr1 APC Cy7	Biolegend	Cat # 108424
CD11b PE	Biolegend	Cat # 101208
CD11b APC Cy7	Biolegend	Cat # 101226
CD3 PE Cy7	Biolegend	Cat # 100220
CD3 FITC	Biolegend	Cat # 152304
CD4 APC	Biolegend	Cat # 100412
CD4 FITC	Biolegend	Cat # 100406
CD8 PerCPCy5.5	Biolegend	Cat # 100734
F4/80 APC	Biolegend	Cat # 123116
Ly6G PE	Biolegend	Cat # 127608
Ly6C FITC	Biolegend	Cat # 128006
Ly6C PerCPCy5.5	Biolegend	Cat # 128012
CD45.1 APC	Biolegend	Cat # 110714
CD45.1 PE-CF594	BD Biosciences	Cat # 562452
CD45.2 BV711	Biolegend	Cat # 109847
FOXP3 PE	Biolegend	Cat # 126404
CD25 APC Cy7	Biolegend	Cat # 101918
IL-17A APC Cy7	Biolegend	Cat # 506940
IFN-γ BV605	Biolegend	Cat # 505840
IL-1β (Pro-form)	eBiosciences	Cat #12-7114-82
TNF-α BV785	Biolegend	Cat # 506341
IL-6 APC	Biolegend	Cat # 504508
G-CSF AF700	R&D Systems	Cat # IC414N-100ug
GM-CSF PE Cy7	Biolegend	Cat # 505412
CD121a (IL-1R, Type I/p80) APC	Biolegend	Cat # 113509
HIF-1α PE	eBiosciences	Cat #12-7528-82
p-NF-kB p65 (Ser536) AF647	Cell Signaling	Cat # 4887S
Rat IgG1, k Isotype BV785	Biolegend	Cat # 400443
Rat IgG2a, k Isotype PE Cy7	Biolegend	Cat # 400521
Rat IgG1, k Isotype AF700	Biolegend	Cat # 400420
Armenian Hamster IgG Isotype APC	Biolegend	Cat # 400911
Rat IgG2b, k Isotype PE	Biolegend	Cat # 400608

Rat IgG1, k Isotype PE	Biolegend	Cat # 400407
Rat IgG1, k Isotype BV605	Biolegend	Cat # 400433
Rat IgG1, k Isotype APC Cy7	Biolegend	Cat # 400422
Annexin-V APC	Biolegend	Cat # 640920
Other Reagents		
7-AAD	Biolegend	Cat # 420404
Annexin-V-Binding Buffer	Biolegend	Cat # 422201
DAPI	Sigma-Aldrich	Cat # D9542-1MG
Cyto-Fast Fix/Perm Buffer Set	Biolegend	Cat # 426803
FOXP3 Fix/Perm Buffer Set (4X)	Biolegend	Cat # 421403
