# Supplementary material for: Ref-1 drives ulcerative colitis induced systemic defects in hematopoietic cells
Source: Commun Biol. 2026 Mar 19;9:635. doi: 10.1038/s42003-026-09860-z (PMC13168444; doi:10.1038/s42003-026-09860-z)
Supplement: Supplementary file 2 — Description of Additional Supplementary Materials [file 42003_2026_9860_MOESM2_ESM.pdf]

Description of Additional Supplementary Files

File name: Supplementary Data 1

Description: The numerical source data for all graphs in the manuscript
